# Biomechanical Effects of Platform Diameter and Screw Length in an Abutment-Free Tissue-Level Implant System Compared with a Ti-Base Configuration: 3D Finite Element Analysis

**DOI:** 10.3390/jfb17010019

**Published:** 2025-12-26

**Authors:** Aliona Dodi, Alecsandru Ionescu, Mihaela Anca Marin, Emil Nuțu, Vlad Gabriel Vasilescu, Ana Maria Cristina Țâncu, Toma Lucian Ciocan, Marina Imre

**Affiliations:** 1 Discipline of Prosthodontics, Faculty of Dentistry, Carol Davila University of Medicine and Pharmacy, 37 Dionisie Lupu Street, District 2, 020021 Bucharest, Romania; aliona.dodi@drd.umfcd.ro (A.D.); mihaela.marin@umfcd.ro (M.A.M.); anamaria.tancu@umfcd.ro (A.M.C.Ț.); marina.imre@umfcd.ro (M.I.); 2 Pedodontics Department, Faculty of Dentistry, Carol Davila University of Medicine and Pharmacy, 37 Dionisie Lupu Street, District 2, 0200221 Bucharest, Romania; 3 Department of Strength of Materials, Faculty of Industrial Engineering and Robotics, National University of Science and Technology “Politehnica” Bucharest, 313 Splaiul Independentei, CD building, District 6, 060042 Bucharest, Romania; emil.nutu@gmail.com; 4 Romanian Research and Development Institute for Gas Turbines COMOTI, 220D Iuliu Maniu Blvd., District 6, 061126 Bucharest, Romania; 5 Discipline of Dental Prosthesis Technology, Faculty of Dentistry, Carol Davila University of Medicine and Pharmacy, Dionisie Lupu Street, No. 37, District 2, 020021 Bucharest, Romania; lucian.ciocan@umfcd.ro

**Keywords:** dental implants, tissue-level, abutment-free, direct-to-implant, finite element analysis, oblique loading, screw preload, zirconia crown

## Abstract

This finite element analysis compared a tissue-level implant with an engaging Ti-base to abutment-free, direct-to-implant, tissue-level configurations (3.7 mm and 4.5 mm platforms; short and long retention screws) to examine how platform width and screw length influence stresses under axial and oblique loads. Five configurations were modeled with identical materials and boundary conditions. Screw preload corresponding to a tightening torque of 35 N·cm was applied in the first step, followed by either a 400 N axial load or a 300 N at 30°. Oblique loading dominated the mechanical response, increasing stresses relative to axial loading and concentrating them at the implant neck and first thread, as well as at the crown screw-access and antirotation regions. Under oblique loads, the 3.7 mm platform implant showed the highest stresses, whereas the 4.5 mm platform implant was comparable to or slightly less stressed than the Ti-base configuration, whose peaks remained confined to a small internal recess. Crown stresses remained localized around the antirotation features, while the composite layer bore negligible load. Within the limitations of this numerical model, abutment-free, direct-to-implant workflows may achieve biomechanical performance comparable to Ti-base solutions if platform and screw selection are aligned with the occlusal scheme, but ISO-style fatigue testing and experimental or clinical validation are required.

## 1. Introduction

Finite element analysis (FEA) is increasingly becoming an important instrument in implant dentistry, used to map the path of the occlusal forces along the crown–abutment–implant complex and into peri-implant bone, identifying regions susceptible to mechanical complications and marginal bone remodeling under clinically relevant loading conditions [1,2]. Through multiple implant designs and systems, inclined, nonaxial loading consistently produces higher stress than axial loading, with superior stress at the implant neck and around connection features of the superstructure. These mechanical patterns are consistent with clinical observations that early marginal bone remodeling tends to be crestal to the first threads and emphasize the biomechanical importance of the neck area [2,3,4].

In finite element analysis, stress (and, when relevant, strain) fields are primary descriptors of the mechanical response of a system, providing insight into load transfer, deformation patterns, and the localization of mechanically demanding regions. Strain is also relevant because it reflects deformation levels that relate to joint stability and the mechanical environment of peri-implant tissues. Because functional loading produces multiaxial stress states, strength hypotheses are used to reduce tensor stresses to scalar measures that can be compared across designs and interpreted in relation to material behavior. In this study, von Mises equivalent stress was used for metallic components (Ti-6Al-4V), while crown behavior was primarily evaluated using maximum principal stress (σ_1_) to capture tensile-driven response in zirconia; von Mises maps for the crown are included as supplementary comparative visual descriptors.

Two design parameters are the foundation of most of the clinical–mechanical performance: vertical location of the implant–prosthesis junction (tissue-level or bone-level) and presence or absence of an abutment. Tissue-level implants locate the microgap above the bone crest and provide a more substantial transmucosal collar, consequently decreasing crown-height space effectively and increasing connection diameter, theoretically reducing neck bending under oblique loading [2,5]. Bone-level implants locate the junction at or below the bone crest. Abutment form, adaptation, and preload are important factors for sealing and load-sharing in such a scenario, and any junctional micromotions and microgaps become highly biomechanically and biologically relevant [2,4]. Comparative modeling under harmonized loads has been shown to indicate that, with equal variables, tissue-level implants cause less stress at the cortical level compared to bone-level configurations, depending on load angle, crown height, and platform size [2,6,7,8,9,10]. Platform switching or using an abutment that is narrower than the implant platform has been reported to transmit forces more axially and away from the crest, minimizing cortical peaks under oblique vectors [2,11,12,13,14,15].

The stress field is also managed by the microdesign of the connection. Internal conical (Morse cone) connections have been reported to demonstrate better sealing and lower micromotion than external hex. Screw-retained reconstructions are based on preload and friction at interfaces to provide joint stability. Numerical and in vitro studies indicate that the loss of removal torque under cyclic or tilted conditions relates to interface geometry, surface finish, torque application method, and misalignment [16,17,18,19,20]. Using monolithic zirconia crowns cemented to a Ti-base creates two additional interfaces: implant–Ti-base and Ti-base–crown, with their respective susceptibility to microgaps and micromotion [9,21,22,23]. Clinically, the switch from cemented to screw-retained superstructures is led by problems with the cement residue causing peri-implant inflammation. Therefore, removal of the luting step avoids biologic risk and reduces superstructure removal and maintenance complexity [3,4,9].

In controlled environments, preload and connection geometry determine the path of the load, and the directions of the forces are more important than magnitude in the control of peak stresses [2,24,25]. Intra-brand FEA comparisons of a tissue-level, abutment-free implant, with a direct-to-implant crown design, and a classic tissue-level implant with a Ti-base approach, under identical numerical hypotheses that explicitly address platform width (P37 vs. P45) and screw length (short vs. long) using standardized axial/oblique loads remain uncommon [1,26].

Abutment choice is another clinically relevant variable that can alter the load path in implant-supported single crowns. In a 3D finite element analysis comparing titanium, zirconia, and hybrid zirconia assemblies supported by a titanium base, Pumnil et al. showed that incorporating a Ti-base can redistribute stresses by absorbing part of the load within the Ti-base, while the screw and/or implant may remain critical regions depending on the configuration [27]. These findings support the need to analyze how changing from a Ti-base-mediated connection to an abutment-free, direct-to-implant design may shift stress concentrations within the prosthetic components and implant neck.

Abutment-free (direct-to-implant) restorations remove the abutment altogether, directly securing the monolithic zirconia crown to the implant via a dedicated prosthetic screw and antirotation components integrated in the implant head. The logic is to reduce component count, avoid cement, and have fewer interfaces vulnerable to microleakage [3,28]. But this setup alters how load is scattered between the screw, crown, and implant fixture. In the absence of a Ti-base buffer, extra stress might be transferred to the adjacent zirconia of the screw-access/antirotation details and to the implant–screw complex itself, particularly under oblique loading [29,30]. To our knowledge, for peer-reviewed 3D FEA comparison of an abutment-free tissue-level design versus abutment-based systems, the abutment-free model concentrated more von Mises stresses onto the fixture, screw, and cortical bone-under predominantly oblique and cyclic-static loading conditions, while a Ti-Zr alloy (Roxolid) abutment-based system bore the lowest total stresses [1].

Material selection is linked with connection geometry. Ti-6Al-4V (grade 5) remains a reference for strength, corrosion, and fatigue resistance, although binary Ti-Zr alloys may have better fatigue strengths at similar stiffness [31,32,33,34]. Monolithic zirconia now prevails in restorations because of its high flexural strength and chipping resistance. However, FEA shows that the enhancement of the framework’s stiffness leads to increased local stress concentrations, despite a simultaneous reduction in global deformation. The extent of this trade-off is a function of the geometry of the connections and the orientation of loading [25,35,36,37]. In screw-retained monolithic zirconia, careful management of geometry around the screw-head seat/countersink and antirotation features is critical as these discontinuities are stress concentrators [21,30].

Beyond load size and direction, screw access orientation and crown height can modulate bending moments and local stress concentrations in screw-retained restorations [1,2,38].

Methodological precision is a requirement for valid comparison. Current reviews emphasize that coherent boundary conditions, contact definition, and mesh convergence plots in critical areas for stress (zirconia in the vicinity of the screw-access/antirotation area, implant neck, screw fillet) are essential for inferring configuration differences. Usually, FEA considers homogeneous isotropic bone and bonded osseointegration. The availability of in vitro strain/displacement measurement validation aids interpretation, but even in their absence, like-for-like comparisons under identical numerical assumptions yield high internal validity for ranking stress patterns [24,25,39,40]. In implant dentistry, the International Organization for Standardization (ISO) 14801:2016 provides a helpful reference for worst-case angles and lever arms under dynamic loading; load angle alignment to ISO-like geometries (e.g., 30°) improves comparability and directs fatigue-risk interpretation [38,41].

Within this landscape, TRI’s Matrix platform (TRI Dental Implants Int. AG, Hünenberg, Switzerland) represents an abutment-free, tissue-level, digitally oriented design that allows monolithic zirconia restorations to be screwed directly to the implant without a Ti-base or cement. In this work, the TRI Matrix is compared to an intra-brand control, the TRI Octa tissue-level implant (TRI Dental Implants Int. AG, Hünenberg, Switzerland), restored with an Engaging Ti-base and monolithic zirconia crown. For clinically relevant design levers examination with the abutment-free concept, we modify two prosthetic platforms (P37 and P45) and, for both, short and long prosthetic screws [42,43].

Aim: To compare tissue-level implant with an Engaging Ti-base (TRI Octa) vs. abutment-free, direct-to-implant designs (TRI Matrix; P37/P45; short/long screw, TRI Dental Implants Int. AG, Hünenberg, Switzerland) under the same, standardized two-step protocol (35 N·cm torque to assess preload; 400 N axial; 300 N at 30°), to identify where and how stresses concentrate in the fixture, Ti-base abutment, and zirconia crown.

Research questions:

1. Does the absence of the abutment (TRI Matrix) change the position and value of peak stresses compared to TRI Octa under the same loading?
2. Within the abutment-free design, does platform width (P37 vs. P45) decrease implant-neck peaks when subjected to loading?
3. Does screw length (short vs. long) alter the load path (platform-level sharing vs. compression beneath the screw head) and stress concentrations adjacent to the access/antirotation features?

We employed linear-elastic models under the same loading conditions and applied an ISO-like 30° oblique loading to model a worst-case lever arm. We did not estimate fatigue life, and the stresses were interpreted comparatively to find critical components/regions. Modeling details are given in Section 2.

## 2. Materials and Methods

This study presents a three-dimensional finite element (FEA) comparative analysis of stress distribution within the restorative framework of a tissue-level TRI^®^ Octa implant with an engaging Ti-base versus abutment-free TRI^®^ Matrix configurations, for two prosthetic platforms (P37, P45) and two retention-screw lengths (short, long). Hypotheses and research questions are detailed in the Introduction. We investigate whether eliminating the prosthetic abutment alters the size and location of stresses in the implant, Ti-base abutment, and the prosthetic crown, and if these responses are influenced by platform size and screw length under identical axial and oblique loading. Five distinct configurations of the TRI^®^ Octa and TRI^®^ Matrix systems are assessed to highlight differences in biomechanical behavior under clinically representative loading and to discuss their clinical implications.

For the finite element analysis, the biomechanical assemblies were defined and simplified in SpaceClaim and DesignModeler in Ansys Workbench 2024 R1 (Ansys Workbench 2024 R1; Ansys, Inc., Canonsburg, PA, USA) and then imported into Ansys Mechanical 2024 R1 for meshing, solution, and post-processing.

All values are expressed in the International System of Units (SI) [44]. The notation used is E (Young’s modulus), ν (Poisson’s ratio), σ_y_ (yield strength), σ_u_ (ultimate strength), μ (coefficient of friction), and σ_1_ (maximum principal stress). Units: lengths in mm, areas in mm^2^, forces in N, torques in N·cm, stresses in MPa (GPa for moduli), and angles in degrees (°).

Boundary conditions and the loading scheme are described in the subsequent sections.

**Figure 1:**
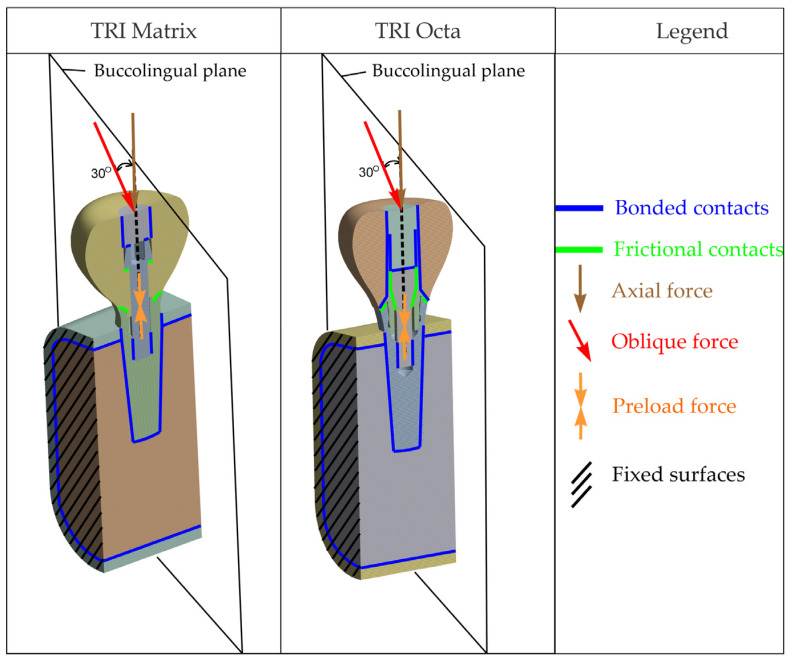
Overview of the complete finite element assembly, boundary conditions and loading scheme. The black dashed line denotes the implant (screw-access) axis used as the reference for load application (axial and 30° oblique) and section orientation.

**Figure 2:**
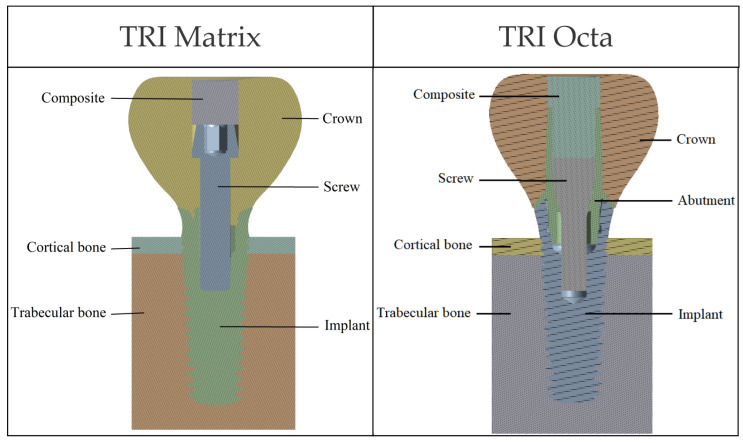
Geometric components included in the model: TRI Matrix (without prosthetic abutment) and TRI Octa (with Ti-base Engaging).

**Figure 3:**
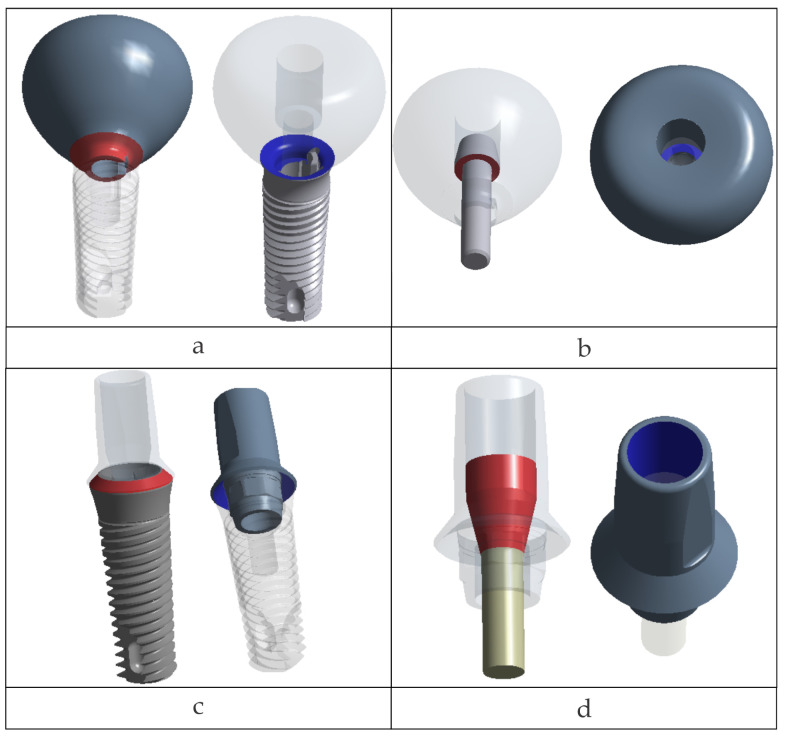
Nonlinear (frictional) contacts: crown–TRI Matrix implant platform (**a**); crown–retention screw in Matrix (**b**); Ti-base–TRI Octa implant platform (**c**); retention screw–Ti-base in TRI Octa (**d**).

**Figure 4:**
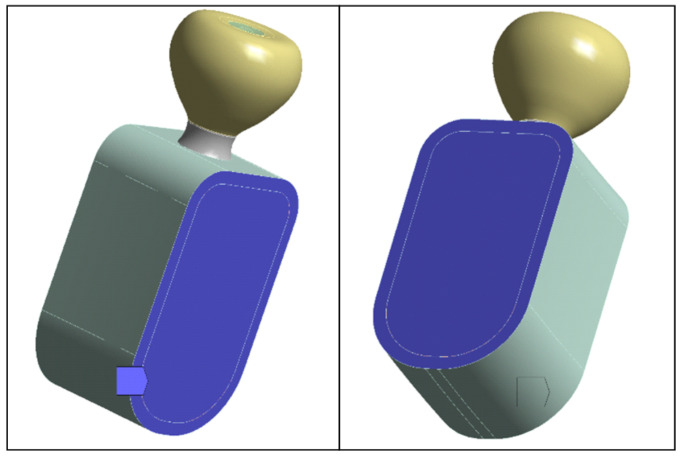
Surfaces on which nodal displacements were constrained.

**Figure 5:**
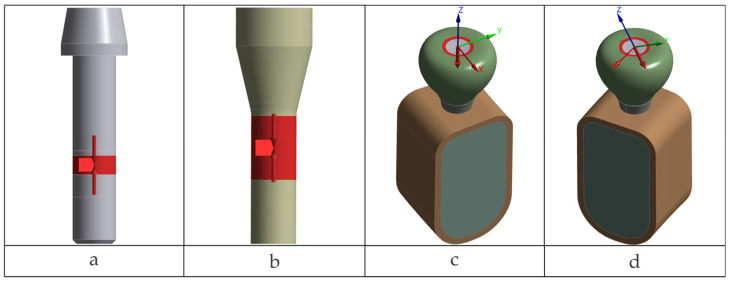
Loading: screw preload (**a**,**b**); axial occlusal force (**c**); oblique occlusal force (**d**).

**Figure 6:**
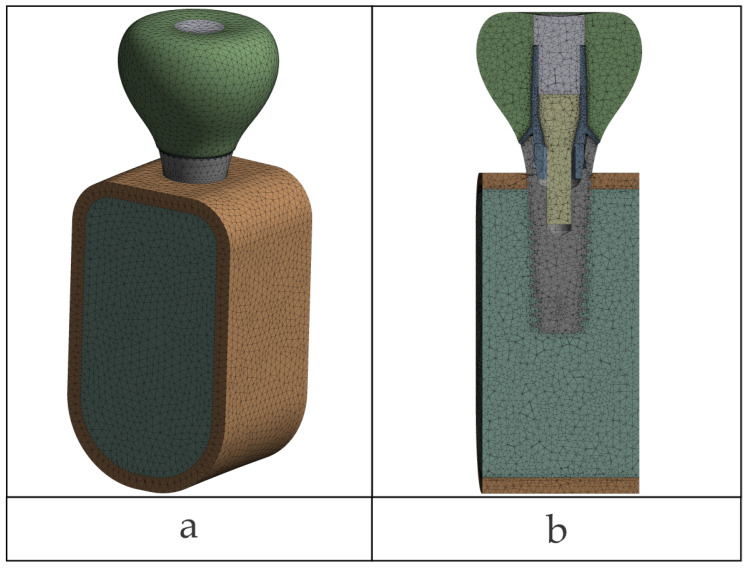
Finite element discretization with higher order tetrahedral elements: (**a**) overall view; (**b**) cross-sectional view.

Presented in Figure 1 are general overviews of the two dental implant assembly models, including boundary and interface conditions. Further details are provided in Figure 2, Figure 3, Figure 4, Figure 5 and Figure 6 and corresponding comments.

### 2.1. CAD Models and Analyzed Configurations

CAD models of the components were provided as STEP files based on manufacturer- geometry, with declared simplifications of details not relevant to the mechanical objectives. The implants investigated were TRI^®^ Octa (tissue-level) and TRI^®^ Matrix (abutment-free, tissue-level/multilevel), manufactured by TRI Dental Implants Int. AG, Hünenberg, Switzerland [42,43]. Five scenarios were investigated with harmonized materials, boundary conditions, contacts, retention-screw torque to assess preload (35 N·cm), and loading schemes between configurations.

The five analyzed configurations are summarized in Table 1 (including the TRI Octa + Engaging Ti-base control), along with the OEM part numbers (SKUs).

For all scenarios, loading conditions included 400 N axial and 300 N at 30° applied on a specific occlusal patch (described in the “Loads” subsection).

Dental crowns were created using CAD tools (SpaceClaim) as axially symmetric solid volumes of the desired size for the corresponding implants. Screw and implant threads (within screw engagement regions) were not modeled, as thread-level detail is beyond the scope of this work. We adopted a smooth-body representation for screws and evaluated their response at the body level, forgoing the resolution of local thread geometry. The composite volume used to seal the crown’s access channel was generated in the remaining space.

The bone block was generated in simplified form, based on data from the literature [45], to mimic a mandibular volumetric segment corresponding to a single tooth. The cortex thickness was set at 1 mm. Figure 2 illustrates the components of the two biomechanical assemblies: without a prosthetic abutment (TRI Matrix) and with a prosthetic abutment (TRI Octa).

### 2.2. Material Properties

All materials were considered homogeneous, isotropic, and linear elastic. Zirconia (3Y-TZP) was treated as a brittle, linear-elastic ceramic; plastic behavior was not assumed. The relevant material parameters are E (Young’s modulus) and ν (Poisson’s ratio), which describe stiffness in the mathematical model. For the interpretation of results, we used strength parameters: σ_y_ (yield strength) and σ_u_ (ultimate strength; flexural/ultimate for zirconia). The values used are summarized in Table 2; ν = 0.30 was assigned to all materials. Properties of Ti-6Al-4V and ZrO_2_ 3Y-TZP were obtained from standards and technical data sheets [34,46,47,48], from the experimental literature [49] for the composite material, and tissue-level properties were used for cortical and trabecular bone [50,51,52].

### 2.3. Mechanical Interfaces

Two types of interfaces were defined between assembly components:

- Nonlinear, frictional surface-to-surface contacts allowing relative sliding, with a friction coefficient of μ = 0.10 [56,57,58,59,60];
- Bonded contacts that enforce zero relative motion across the surfaces.

In Ansys Mechanical, for bonded contacts, the mating surfaces are tied; thus, no separation or relative sliding is permitted.

The contact definitions adopted for each configuration are summarized in Table 3.

In the Octa model, a multipoint constraint (MPC) linked all degrees of freedom along the antirotational features between the implant and Ti-base. The remaining implant–Ti-base interface was modeled as a frictional contact (μ = 0.10).

The resulting nonlinear (frictional) contact regions are illustrated in Figure 3.

### 2.4. Boundary Conditions

To simulate the contact of the mandibular segment with bone segments excluded from the model, fixed-displacement boundary conditions were applied on the section faces shown in Figure 4. All translational degrees of freedom were constrained on these surfaces. These constraints are equivalent to a local rigid anchorage and may induce artificial (nonphysiological) stress concentrations in the vicinity of the blocked faces. But because the model is designed to act as a mechanical support to the implant and the study focuses on the implant–retention screw–crown assembly (rather than stresses in bone tissue), such an idealization is appropriate. Post-processing was carried out in regions of interest distant from fixed boundaries to limit edge effects.

### 2.5. Preload Application (Step 1)

Screw preload was modeled as an internal preload (bolt load) caused by torque wrench tightening (tightening torque T = 35 N·cm, as recommended by the manufacturer). An axial force of F = 812 N was induced in the shank of the retention screw (Figure 5a,b), equivalent to T = 35 N·cm. The conversion followed the procedure described in Tohnichi, Torque Handbook (Tohnichi America Corporation, Buffalo Grove, IL, USA) [61]. Preload was applied in Step 1; occlusal forces were superimposed in Step 2 on the preloaded state. The same axial preload (F = 812 N) was used across all five cases (Octa and Matrix P45 and P37; short/long retention screw), so that response variations were attributed to the assembly structure and contact conditions rather than to the preload variation.

### 2.6. Loads

Functional loads were applied to the crown as either a 400 N axial force or a 300 N oblique load at 30° distributed over a circular occlusal patch (radius R = 1.25 mm, area ≈ 5 mm^2^ constrained by occlusal morphology) centered on the screw-access axis. The load was applied as a remote force with rigid coupling to the nodes of the patch (the red circular surface in Figure 5), ensuring a uniform distribution equivalent to a single resultant acting at the patch centroid. A finite occlusal patch was used to avoid singularities and to normalize the comparison among the five configurations. For this idealized loading protocol, the exact area is not relevant for the analysis.

A total of 400 N applied along the implant axis was used for axial occlusal loading [62] (Figure 5c), while for oblique loading (Figure 5d), an amplitude of 300 N inclined at 30° in the buccolingual plane was used [63], representing a severe but clinically plausible scenario. The loads of 400 N (axial) and 300 N at 30° (oblique) were chosen to reflect realistic chewing forces. Oblique forces generate a much larger bending moment than axial ones, even at lower magnitudes. For this reason, a slightly smaller oblique load is commonly used in implant FEA to reproduce a comparable level of mechanical challenge and a clinically plausible representation of para-axial loading.

The analysis was performed in two steps. In Step 1, bolt preload was applied to the retention screw alone, to simulate the preloaded situation in the implant, screw, and crown. In Step 2, occlusal forces were superimposed on this preloaded situation. The simulation was conducted under static (quasi-static) conditions; cyclic loading was not simulated, because the aim was to make a comparison of stress distributions rather than durability estimates.

### 2.7. Meshing and Local Refinements

Quadratic tetrahedral finite elements (ANSYS SOLID187, 10-node, Ansys Workbench 2024 R1, Ansys, Inc., Canonsburg, PA, USA) were used. The global mesh element size was specified as 0.5 mm (edge length) and also set as the maximum element size control for meshing. Local refinements were performed to interfaces, down to 0.06 mm in nonlinear contact and 0.20 mm in linear/bonded regions (see the Section 2.3). Global minimum element size was not defined; minimum sizes were imposed using local sizing in designated areas. The element-size growth rate between refined zones and the global mesh was set to 1.2 for all models.

Refinement was manual with user-imposed local/face sizing on surfaces close to contact interfaces and where there were steep stress gradients; automatic proximity- or curvature-based size functions were not employed. On the occlusal patch, the mesh was smoothed out with manual face sizing to represent the load distribution accurately. No target size was assigned to the patch elements; refinement was in the same limits (0.06 mm near nonlinear contacts; 0.20 mm in bonded or moderate-gradient regions).

A mesh detail value for Octa configuration is presented in Figure 6a and is typical of all of the models; interface refinements are shown in Figure 6b. Mesh quality was verified in Ansys Mechanical using the Jacobian ratio (corner nodes). Automatic meshing used the mechanical-aggressive option with checks for distorted elements; none were found. The Jacobian ratio (corner nodes) was greater than the minimum of 0.03 in all models. Because of the practical equivalence of most mesh-quality measures, only Jacobian ratio was reported, without additional statistics for skewness, aspect ratio, or Jacobian.

Final mesh statistics in each case are reported in Table 4.

### 2.8. Solution Settings

Simulations were executed and post-processed in Ansys Mechanical 2024 R1 (Ansys, Inc., Canonsburg, PA, USA) using the nonlinear implicit Newton–Raphson-method-based solver. Analysis was nonlinear quasi-static in the small-displacement regime.

Nonlinear contacts were defined as surface-to-surface via the augmented Lagrange algorithm. Small sliding was enabled; normal stiffness was program controlled (automatic) and initial gaps in critical interfaces were closed prior to solution. Advanced contact parameters (normal and tangential stiffness, contact detection method, penetration tolerances, and update rules) were defined as program controlled (Ansys defaults) without additional customization.

Loading was incremented in user-imposed substeps to increase solution convergence. A total of 10 initial substeps per step (preload and occlusal loading) were used, with a minimum of 5 and a maximum of 30 substeps with program-controlled Newton–Raphson convergence tolerances.

All simulations were run on a workstation equipped with two Intel^®^ Xeon^®^ E5-2630 v3 processors (Intel Corporation, Santa Clara, CA, USA; 2.40 GHz) and 48 GB RAM.

### 2.9. Convergence Study

For mesh convergence testing, successive analyses were run with different local element size within regions of interest (nonlinear contact interfaces and steep stress gradient regions). Model-dependent singularities (edges/corners) were disregarded because they could produce nonrepresentative theoretical maxima. Stresses were computed as nodal-averaged values. Stabilization of von Mises stresses within regions of interest was ensured after final refinement. Convergence was considered achieved when both the location of peak-stress zones and the order of magnitude of von Mises values in the regions of interest remained stable between the last two refinements.

The boundary conditions and loading protocols are aligned with standard FEA in implantology [24,41], and the spatial distribution and order of magnitude of the peak stresses obtained in our configurations are harmonious with recent reports, supporting the numerical plausibility of the models [16,17,25].

### 2.10. Regions of Interest and Post-Processing

Result extraction was aimed at clinically and mechanically relevant areas: the implant neck/platform; the geometry of the antirotational feature and screw-access channel of the crown; and the configuration-specific nonlinear contact interfaces (crown–implant and crown–retaining screw for TRI^®^ Matrix; retaining screw–abutment and abutment–implant for TRI^®^ Octa). Fixed interfaces were considered to be perfectly bonded, linear connections without slip or separation, e.g., the crown–abutment joint in TRI Octa and all other permanent joints.

For metallic components, von Mises equivalent stress was selected as a standard reduced-stress metric for ductile Ti alloys under multiaxial loading; alternative criteria (e.g., Tresca) could also be used without changing the qualitative comparison of hotspot locations across configurations. Shear stress components were not reported as standalone outcomes; their contribution in metallic parts is reflected in the von Mises equivalent stress.

For the ZrO_2_ (3Y-TZP) crown, the tensile/compressive regime was assessed from σ_1_ and σ_3_, supported by σ_1_ maps and principal-stress vector plots. Therefore, zirconia crown comparisons and failure-risk interpretation are based primarily on tensile maximum principal stress (σ_1_) and its location, while von Mises is reported only as a global, qualitative descriptor and is not used as a failure criterion for 3Y-TZP. Predominantly compressive fields were further characterized by the study of principal-stress direction maps. To limit the effect of singularities, stresses were considered in finite areas rather than at isolated points. Stresses were retrieved as nodally averaged values (nodal averaging ON) and results are provided separately for Step 1 (preload) and Step 2 (preload + occlusal loading, axial and oblique), with values retrieved at the end of each step.

### 2.11. Safety Factor, Allowable Stress, and Reporting Thresholds

**Figure 7:**
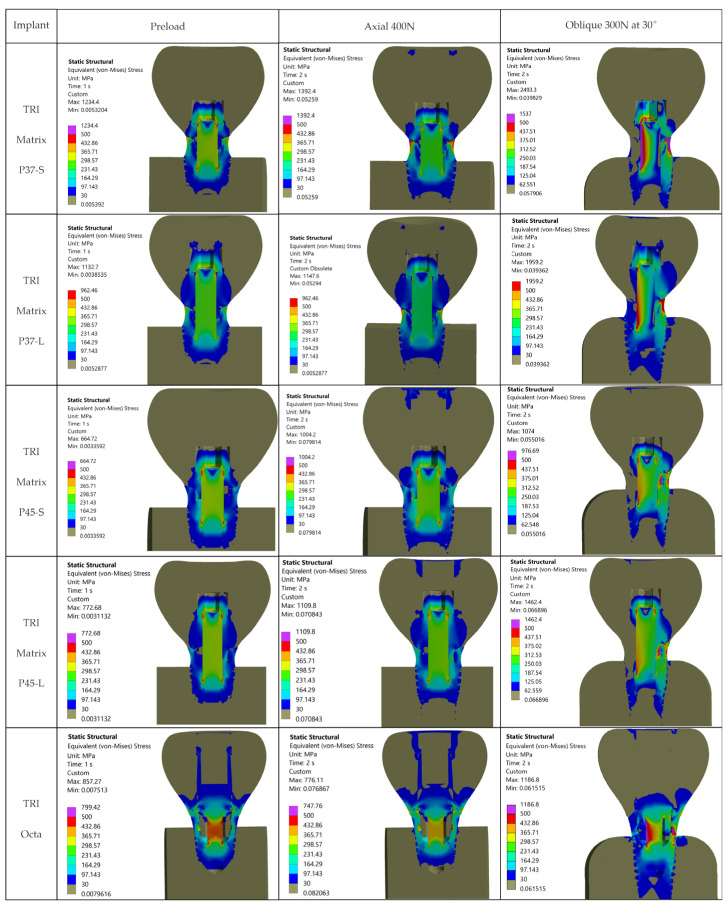
von Mises stress maps for the five TRI configurations (rows) and loading steps (columns: screw preload; axial 400 N; oblique 300 N at 30°). Unified scale, 30–500 MPa; values below/above the window in separate bands. Hotspots: implant neck/first thread; crown screw-access/antirotation feature.

Metallic components (Ti-6Al-4V) were evaluated against a nominal yield strength of about 800 MPa [47]. We applied a safety factor of 1.6 to define an acceptable stress σ_adm_ = σ_y_/SF = 500 MPa, which was employed for descriptive reporting of metallic component and figure/table captioning. The overview maps (Figure 7) generally presented a 30–500 MPa display window; component-specific maps applied adjusted windows (e.g., crowns capped at 200 MPa; implant/Ti-base views with 500/800 MPa thresholds presented). For zirconia crowns (3Y-TZP), the primary outcome was the maximum principal stress (σ_1_). Flexural/ultimate strength values of around 1.1 GPa served as contextual references, not admissible thresholds [46,48]. Reporting decisions and transparency adhere to guidelines for FEA in implant dentistry [24].

### 2.12. Discretization Statistics

Mesh statistics (the total element and node counts) used for each configuration are provided in Table 4.

## 3. Results

The relevant outcomes for this study are the stress fields in the crown, the implant, and in the prosthetic abutment (Ti-base, TRI Octa only). Figure 7 shows the von Mises stress distributions in the analyzed biomechanical assemblies. The first column corresponds to the state generated by screw preload (Step 1), without occlusal force. The second column corresponds to superimposing an axially oriented occlusal force (400 N) on the preloaded state (Step 2: axial loading), and the third column to Step 2 with an oblique occlusal force (300 N at 30°).

### 3.1. Global Stress Maps-Overview

For graphical representation of stress distributions, eight color bands were used, with the range between 30 MPa and 500 MPa. Values outside these limits were rendered in separate bands, highlighting regions below or above the analyzed window. The lower value, 30 MPa, was chosen as a physiological stress threshold in bone tissue (per Frost’s mechanostat [64]), excluding lower stress volumes from the color. As seen in Figure 7, no relevant stress concentrations appear in bone or in the crown away from the functional interfaces within this window. The upper value, 500 MPa, corresponds to the allowable limit for metallic components (σ_adm_ = 500 MPa), derived using a safety factor (SF) of 1.6 relative to the yield strength of Ti-6Al-4V (see Section 2).

In all five configurations, under axial loading (400 N), stresses in metallic alloy components exceed σ_adm_ = 500 MPa, and some configurations exceed even the yield limit (800 MPa), with variations among configurations (details in Table 5). Applying the oblique load (300 N at 30°) increased stresses greatly, with peaks located at the implant neck/first thread and in the crown screw-access region/antirotation feature of the crown (Figure 7). In the implant, hotspots occur for TRI Matrix P37 under oblique loading, whereas in the crown the largest local peaks occur for TRI Matrix P45 (Figure 7 and Table 6). Peak values are reported for localization only. Comparative interpretation relies on the extent and pattern of the high-stress regions.

**Figure 8:**
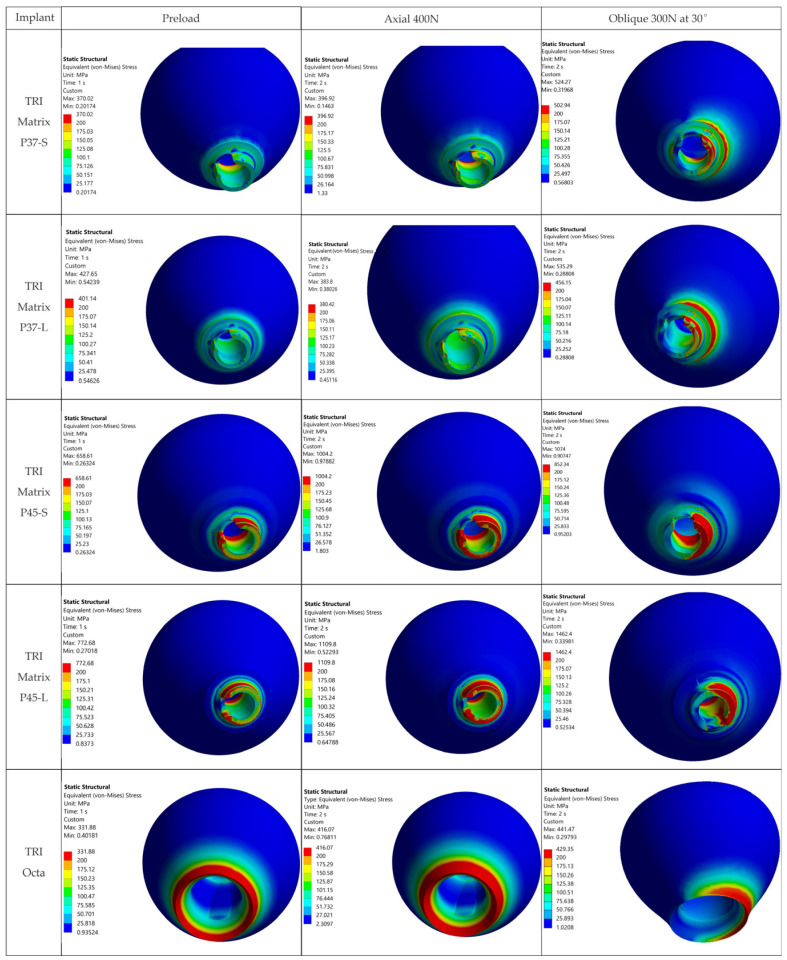
von Mises stresses in the 3Y-TZP crown—views: Step 1: preload in the screw; Step 2: combined preload and axial occlusal force; Step 2: combined preload and occlusal force inclined at 30°.

**Figure 9:**
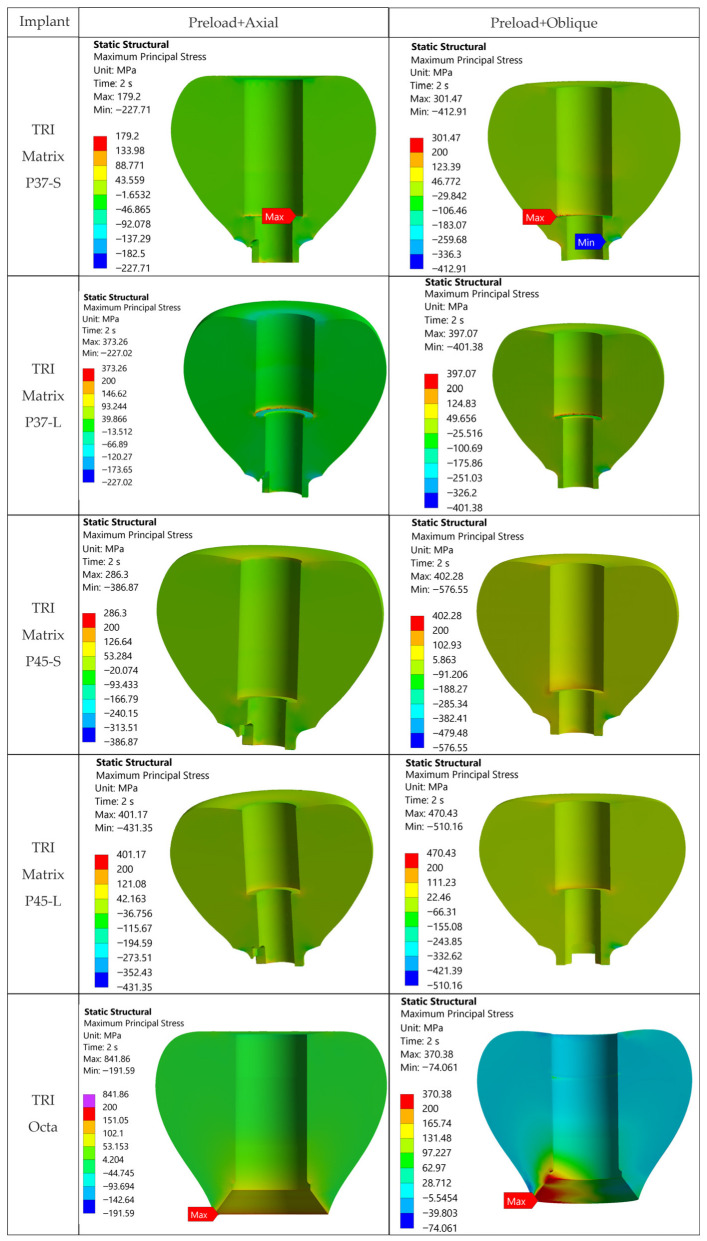
Maximum principal stress maps (σ_1_, MPa) in 3Y-TZP crowns sections. Same unified color map: arrows indicate tensile hotspots.

For 3Y-TZP crowns, von Mises values are reported as a global comparative descriptor. Evaluation on σ_1_ (the primary metric for ceramics) is performed qualitatively using σ_1_ maps and principal-stress vector plots. The value of 500 MPa lies below that derived from flexural/fracture strength (with the same safety factor of 1.6), facilitating identification of over-stressed metallic regions. Maximum principal stress (σ_1_) maps of all five configurations and three loading cases are given in Figure 8 and Figure 9 and Appendix A (Figure A2).

Although the composite has lower mechanical strength than the other components of interest, it does not carry large loads because the occlusal force is transmitted predominantly through the much stiffer crown material. In addition, the loads resulting from screw preload do not materially affect the composite volume.

The maxima by component (implant, crown, and Ti-base for TRI Octa only) are summarized in Table 5, Table 6 and Table 7. Comparisons between TRI Matrix P37 and TRI Matrix P45 indicate differences in stress distribution, detailed in the following subsections.

### 3.2. Numerical Synthesis of Maxima by Component

Table 5, Table 6 and Table 7 compile peak stress values (reported to indicate hotspot locations) for the three loading scenarios (screw preload, axial 400 N, oblique 300 N at 30°) by component (implant, crown, Ti-base). For metallic components we also report σ_max_/σ_adm_, σ_max_/σ_y_ as descriptive flags of proximity to material limits, not failure predictors. Δ% relative to TRI Octa (variation versus the control configuration, for the same component and loading step) is provided for reference only and is not used for between-configuration comparison. For 3Y-TZP crowns, the primary indicator remains σ_1_; von Mises values are used only as a complementary descriptor. Locations of maxima are noted in each table, and display thresholds are specified in Section 2. Comparative interpretation relies on the observed stress-field patterns within the standardized 30–500 MPa window and on the extent of volumes above the relevant thresholds (σ_adm/_σ_y,_ σ_1_) rather than on peak magnitudes.

For the implant (Ti-6Al-4V), the critical case remains oblique loading, 300 N at 30°. Maxima typically occur at the neck/first thread in the TRI Matrix models, and in the internal octagon antirotation interface region in TRI Octa. For screw preload and axial 400 N, values are visibly lower (details in Table 5).

For the 3Y-TZP crown (where σ_vM_ is illustrative), oblique loading consistently produces maxima at the crown’s antirotation feature/screw-access region as localized peaks. Figures for screw preload and axial loading, as well as full comparisons among configurations, are provided in Table 6.

For the Ti-base (TRI Octa), the trend rises with loading severity: 742.0 MPa for screw preload, 748.0 MPa for axial, and 1221.0 MPa for oblique, corresponding to σ_max_/σ_adm_ of 2.44. Peaks are confined to edges/corners over small volumes, a summary is given in Table 7.

Spatial distributions for each scenario are illustrated in Figure 7; the tables condense values and locations for quick reference.

### 3.3. Stress Analysis in the Crowns—Stress Distribution

Figure 8 and Figure 9 present the 3Y-TZP crown stress distributions, using views and sections. The columns have the same meaning as in Figure 7. Evaluation uses von Mises as a comparative descriptor across configurations, while σ_1_ (maximum principal stress) is assessed qualitatively from σ_1_ maps and principal stress vector plots. To enable direct comparison, the σ_1_ fields (sections and views) are given in Figure A2 and Figure 9 on the same MPa color scale.

**Figure A1:**
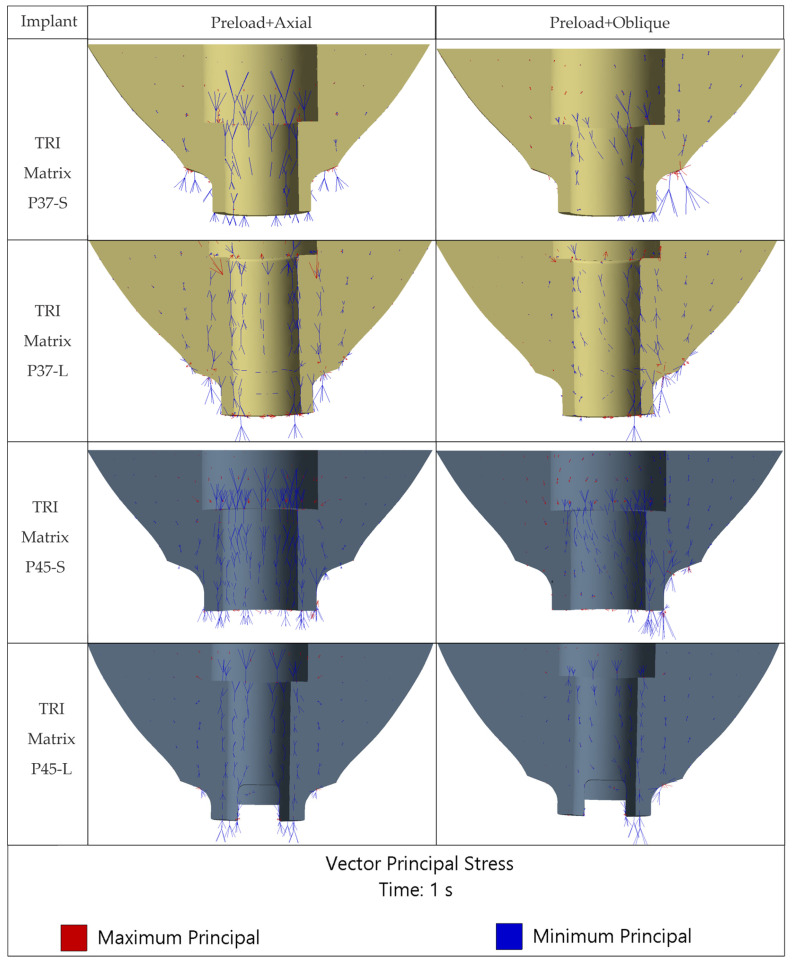
Vectorial representation of principal stresses in cross-section through the 3Y-TZP crown: screw preload: Step 1 (left column) and superimposed inclined occlusal loading: Step 2.

Vector plots of principal stresses (Figure A1) highlight dominant compressive fields in the 3Y-TZP crown after Step 1 (screw preload), with intensification and redistributions after Step 2 (oblique occlusal loading) for the two TRI Matrix implant variants. Tension appears only locally, particularly in oblique scenarios, at levels much lower than compression; consequently, σ_1_ does not control the global static behavior under the investigated conditions, and von Mises reporting is used here as a global comparative descriptor. Comparative interpretation is based on the extent and location of high-stress regions, not on the absolute peak value.

**Figure 10:**
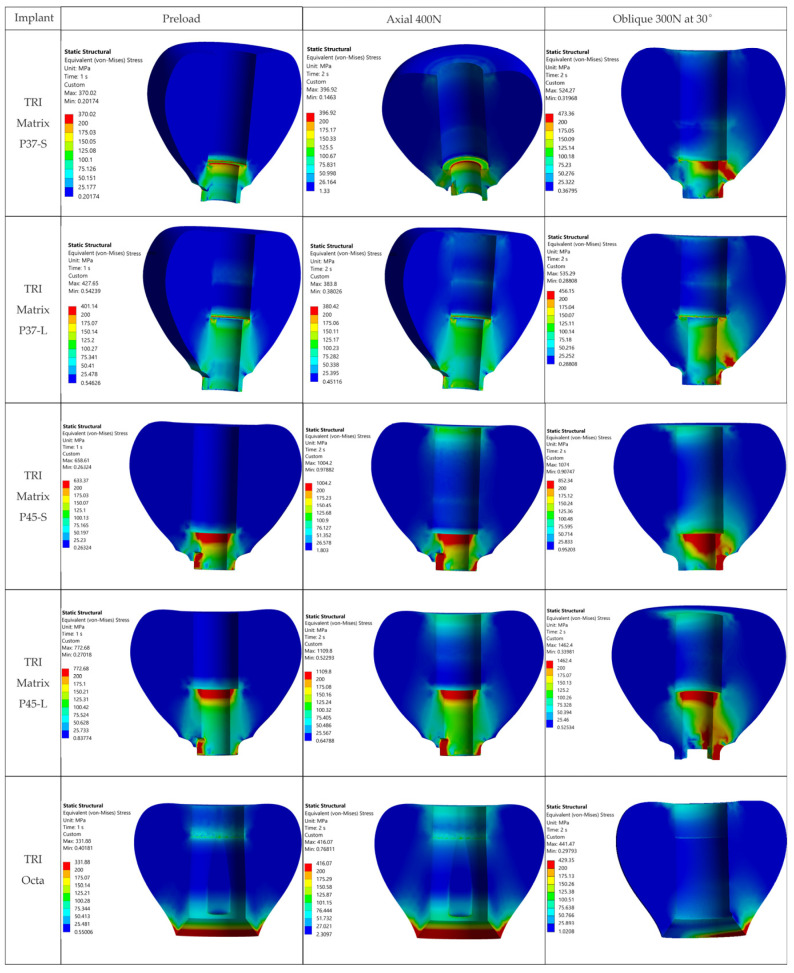
von Mises stresses through the 3Y-TZP crown in section: Step 1: screw preload, Step 2: combined preload and axial occlusal force, Step 2: combined preload and occlusal force inclined at 30°.

For TRI Matrix P37, both with short and long screw, the compressive loads generated by preload (loading Step 1) are distributed preferentially along the periphery of the implant platform rather than through the central region, which yields more uniform stresses. In the long-screw variant, TRI Matrix P37 maintains the distribution on the implant platform, but due to the greater distance between the screw head and the crown’s support surfaces, compressive stresses decrease at the edge of the implant platform, producing a slight increase versus the previous variant in the central region (see also Figure 10 for the quantitative comparison of stresses).

For the TRI Matrix P45, compressive loads concentrate predominantly under the screw head, causing a marked increase in stresses parallel to its axis, concomitant with a decrease at the interface with the implant platform. The effect is more pronounced with the long screw. The observed distribution is coherent with the effect of preload (details in Section 4).

**Figure A2:**
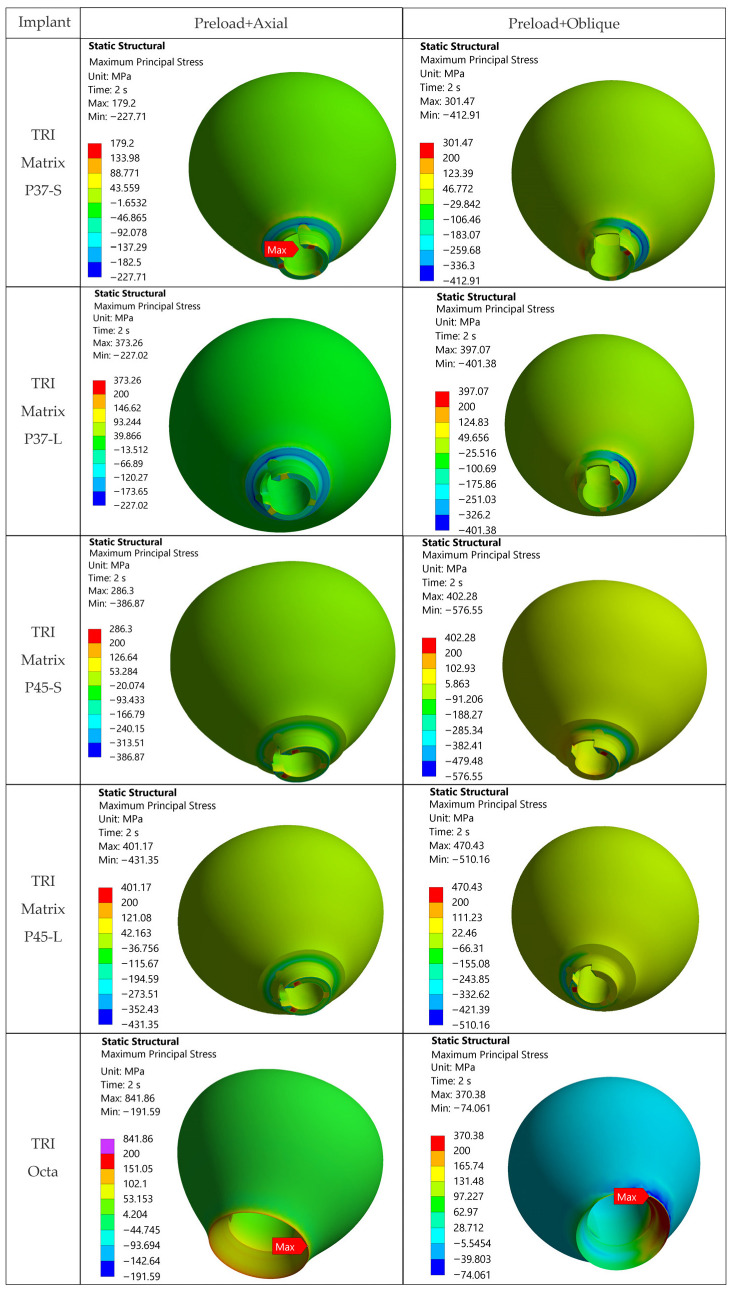
Maximum principal stress (σ_1_, MPa) maps in 3Y-TZP crowns: external views for all five configurations and two loading cases. Unified color map (MPa) in all figures; arrows indicate the antirotation/screw-access hotspot (tension > 0).

Comparison among configurations:

For both screw lengths, TRI Matrix P37 exhibits lower tensile σ_1_ peaks in the crown than TRI Matrix P45 (Figure 9 and Figure A2).

A long screw increases the tendency for stress concentration under the screw head, especially under oblique loading (see Figure 10 for the quantitative comparison).

**Figure A3:**
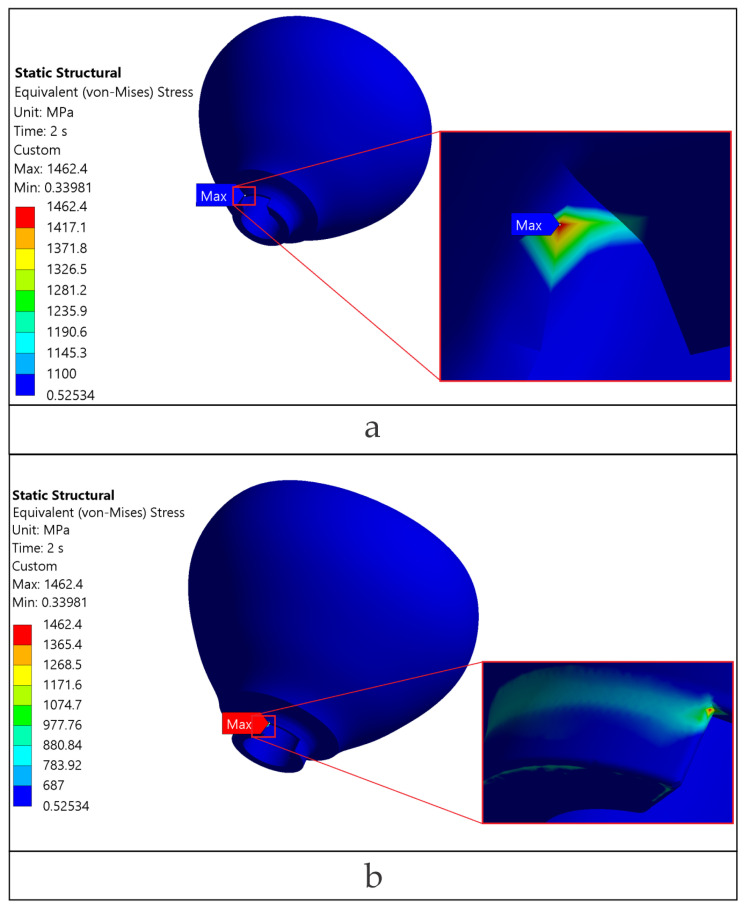
Location of von Mises maxima for TRI Matrix P45-L under occlusal force, inclined loading: 1100 MPa (**a**) and 687 MPa (**b**).

To assess the robustness of the reported peaks, a detailed analysis of crown distributions was performed for configuration TRI Matrix P45-L (the scenario with the highest values). The analysis shows peaks strictly localized at the crown’s antirotation feature, over very small areas (Figure A3), without relevant areal extension into the crown mass. Overall, crown stresses indicate absence of extended tensile regions, and von Mises maxima occur near the antirotation feature (Figure A3). Direct comparison of von Mises with flexural strength (~1100 MPa) is only illustrative. Fracture assessment for ceramics relies on σ_1_ when tensile stresses are predominant. As σ_1_ is low in all the cases (see Figure A1, Figure A2 and Figure 9), and the preload induces a predominantly compressive state into the crown even under oblique occlusal force, tensile-driven failure appears unlikely under the modeled conditions. Therefore, in this study we employed a qualitative analysis of σ_1_ maps and principal-stress directions to support the lack of critical tensile stresses. The ~687 MPa threshold (≈1100/1.6) was used for visualization; exceedances are highly localized under oblique loading and do not involve a critical crown volume. von Mises maxima are recorded for TRI Matrix P45-L (≈1109.8 MPa axial; ≈1462.4 MPa oblique) and for P45-S oblique (≈1074 MPa), but remain localized peaks, compatible with local over-stress without necessarily indicating certain failure and are not used for comparison.

### 3.4. Stress Analysis in Implants—σ_vM_ at the Neck and First Thread

**Figure 11:**
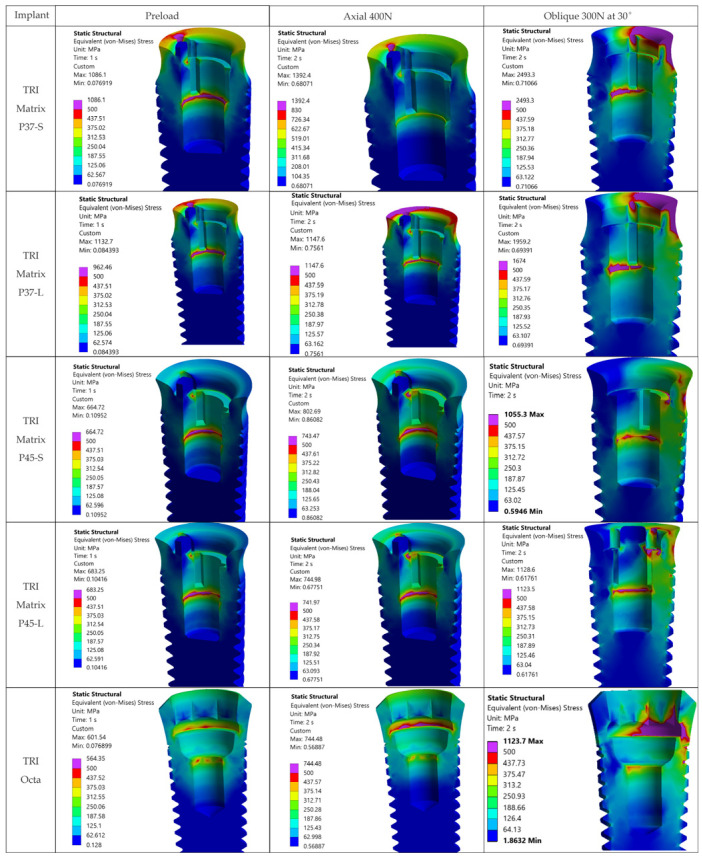
von Mises stresses in the implant, longitudinal section: Step 1: screw preload, Step 2: axial occlusal force, Step 2: occlusal force inclined at 30°.

**Figure A4:**
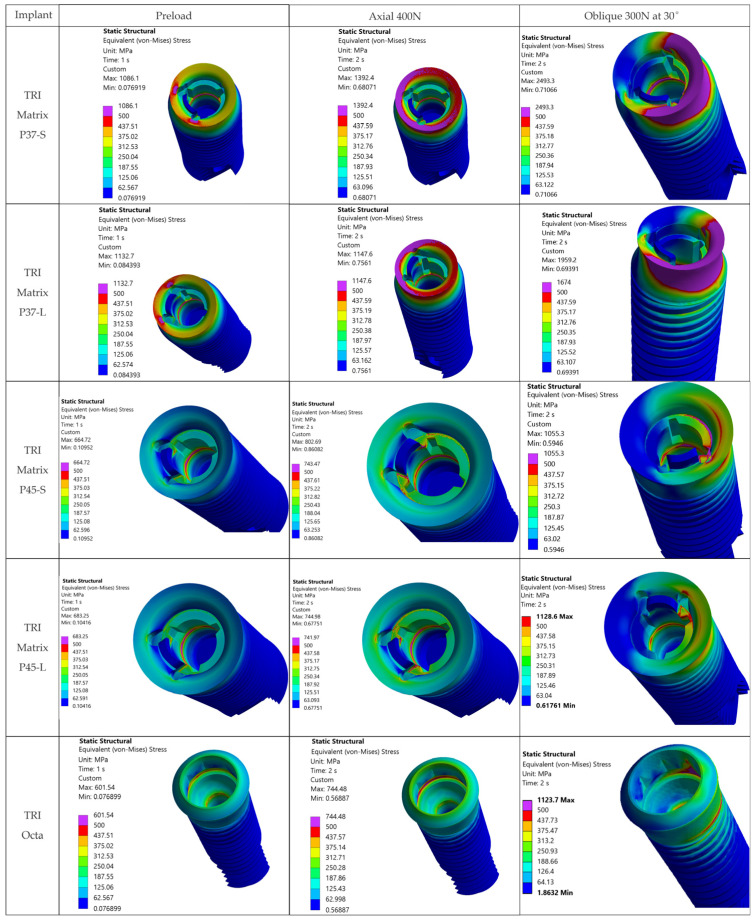
von Mises stresses in the implant-axonometric view: Step 1: screw preload, Step 2: axial occlusal force, Step 2: inclined at 30° occlusal force.

Figure 11 and Figure A4 show von Mises stress distributions in the implants for all five variants, in external view and, respectively, in cross-sections along the implant axis. Comparisons use the σ_adm_ threshold defined in Section 2.

**Figure 12:**
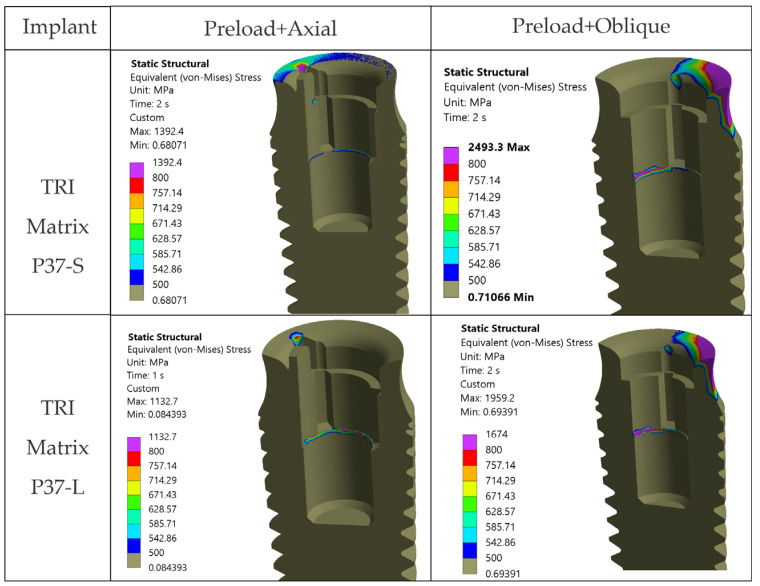
von Mises stresses in the TRI Matrix P37 implant, cross-section: axial occlusal force (left column) and inclined occlusal force (right column).

**TRI Matrix P37**. In TRI Matrix P37, high stresses are identified after retention screw preload for both screw lengths. With the long screw, stresses are higher, exceeding the material yield limit. Axial occlusal loading adds compressive loads on top of those from preload. With the short retention screw, stresses are higher at the neck/first thread because the occlusal load is preferentially transferred on/through the implant platform. Under oblique loading (30°/300 N), σ_adm_ exceedances occur over more extensive volumes. The corresponding cross-sectional von Mises stress distributions for TRI Matrix P37 under axial and oblique occlusal loading are shown in Figure 12.

**Figure 13:**
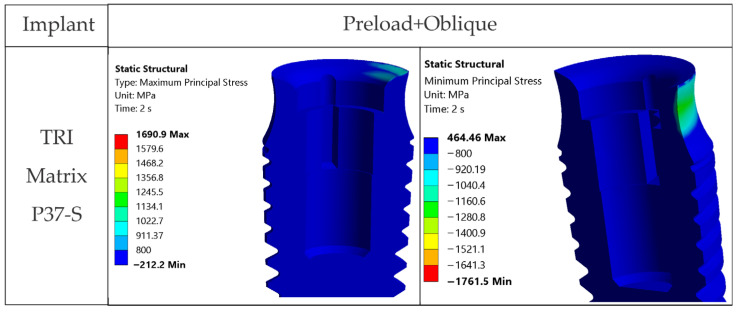
Principal stresses in the TRI Matrix P37-S implant under inclined occlusal loading, cross-section: maximum principal stress (**left**) and minimum principal stress (**right**).

Figure 13 presents maximum and minimum principal stress distributions in the TRI Matrix P37 implant with the short screw, showing tensile states at the platform and compression in the neck region.

**Figure 14:**
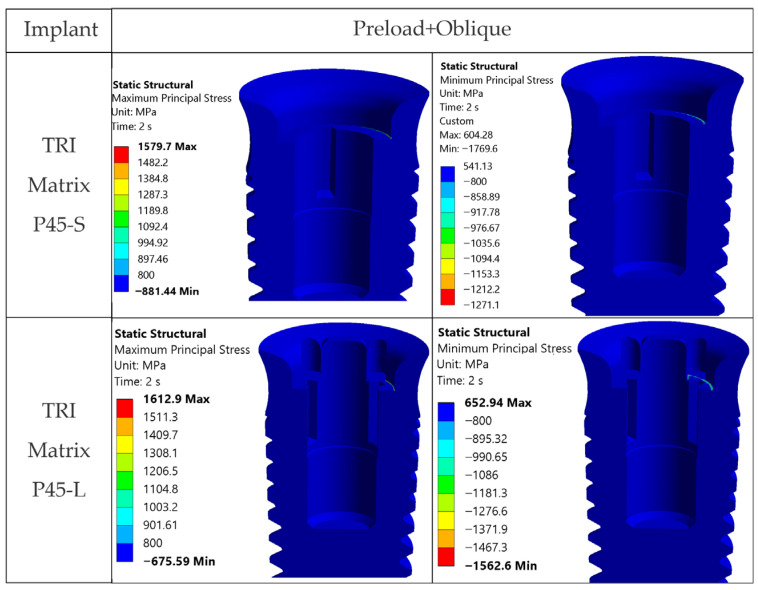
Principal stresses in the TRI Matrix P45 implant under inclined occlusal loading, cross-section: maximum principal stress (**left**) and minimum principal stress (**right**).

**TRI Matrix P45**. From Figure 11 and Figure A4, TRI Matrix P45 shows von Mises stresses above σ_adm_ = 500 MPa across all occlusal loading cases, and even above the yield limit (800 MPa) for the short-screw variant under inclined occlusal force. Figure 14 illustrates this via maximum and minimum principal stresses, which exceed the yield limit extremely locally. In addition, the result may depend on the model, i.e., follow from local geometry and the contact between components.

**Figure 15:**
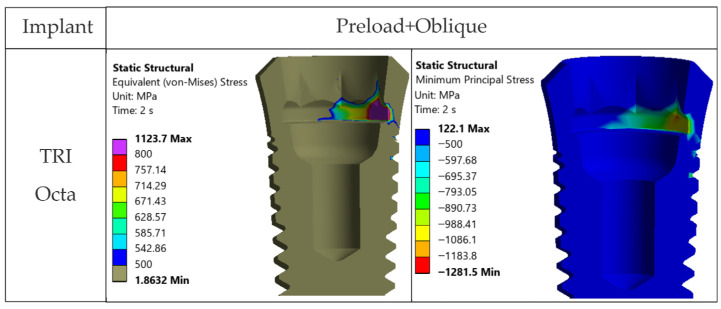
Stresses in the TRI Octa implant under inclined occlusal loading, cross-section: von Mises stresses (**left**) and minimum principal stresses (**right**).

**TRI Octa.** The TRI Octa implant shows the lowest stresses under preload and axial occlusal force (Figure 11 and Figure A4). In these two variants, the allowable stress is exceeded locally, at edge/corner regions, but the values remain below the yield limit (~800 MPa). Under inclined occlusal loading, the Ti-base octagonal antirotation interface acts as a stress concentrator, generating highly localized peaks above the yield limit (Figure 15).

### 3.5. Influence of Retention Screw Length

The analysis shows that screw length influences not only mechanical fixation but also the stress distribution in the assembly. For TRI Matrix P45, for both lengths, loads transfer predominantly under the head of the retention screw, with a more pronounced effect for the long screw, which accentuates axial concentrations and reduces the contribution of the implant platform (see Figure 10, Figure 11, Figure 12 and Figure A4). In contrast, for TRI Matrix P37, for both lengths, the transfer is more uniform at the implant platform. The short screw favors this peripheral discharge; the long screw maintains it, but under oblique loading, a slight increase in loading is observed in the axial region under the screw head compared to the short variant (Figure 10). Consequently, in the crown, von Mises values remain lower for P37 than for P45 for both lengths, while in the implant the differences between lengths reflect the balance between discharge on the platform and transmission under the screw head (Figure 11, Figure 12 and Figure A4).

### 3.6. Stress Analysis in the Prosthetic Abutment—Ti-Base (TRI Octa Only)

**Figure 16:**
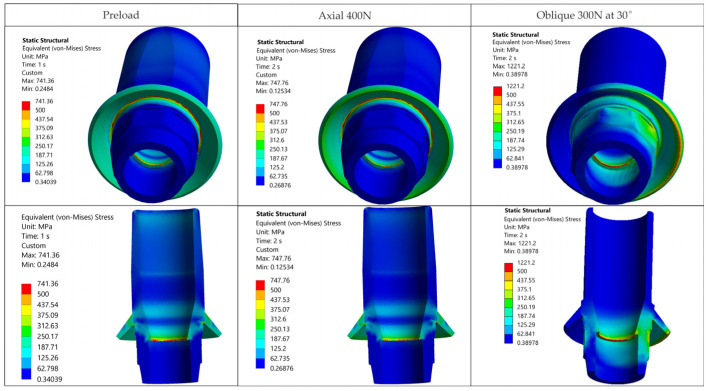
von Mises stresses in the prosthetic abutment: Step 1: screw preload, Step 2: axial occlusal force, Step 2: occlusal force inclined at 30°.

**Figure 17:**
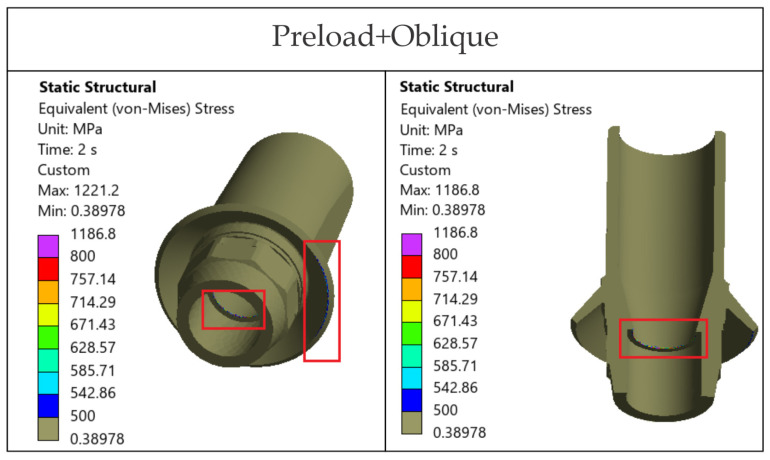
von Mises stresses in the prosthetic abutment with limits of 500 MPa and 800 MPa. Red rectangles indicate the localized edge/corner regions where stresses exceed these thresholds (small volume).

The prosthetic abutment of the TRI Octa system does not indicate a risk of global static failure (Figure 16 and Figure 17). According to Figure 16, von Mises stresses exceed either the allowable stress or the yield limit only at abutment edges. The areas with stresses within or above this interval are extremely small and localized. Under oblique loading, the internal relief of the implant acts as a stress concentrator, generating peaks above the yield limit in this region; the involved surface remains small (Figure 17).

### 3.7. Numerical Verifications and Model Quality Control

Local stress peaks and their orders of magnitude remained in the same regions under successive refinements. Corner/edge singularities were identified and disregarded in interpretation.

Overall, oblique loading at 30° (300 N) generated the reported hotspots for all configurations, increasing stresses both at the implant level and in the crown’s screw-access/antirotation region (Figure 7). In the implant, TRI Matrix P37, particularly the long-screw configuration, exhibited the most severe hotspot at the neck/first thread at cortical bone interface (Table 5). In the crown, TRI Matrix P45, especially with the long screw, concentrated σ_vM_ peaks at the antirotation element, with small volumetric extent (Table 6 and Figure A3). For the Ti-base (TRI Octa), maxima increased with loading severity and remained confined to edges/corners over small volumes (Table 7). The numerical synthesis is provided in Table 5, Table 6 and Table 7 and the corresponding figures.

## 4. Discussion

The resulting distributions demonstrate a trend where stresses relevant to assembly integrity are concentrated at functional interfaces and close to geometric stress raisers. Figure 7 illustrates that, away from crown–implant–screw interfaces, the stress field in the peri-implant bone is under 30 MPa, which is compatible with physiologic loading. For Ti-6Al-4V components we assumed σ_adm_ = 500 MPa [2,3,4]. Relative comparison between configurations relies on stresses recorded in the regions of interest: implant neck/first thread, crown screw-access/antirotation zone, and space beneath screw head (Table 5). Within these areas, we considered representative peak values while disregarding isolated numerical spikes.

The internal accuracy of the model used in this study is reflected in the mesh-convergence verification performed in all regions of interest, where successive local refinements resulted in the stable peak-stress locations and consistent orders of magnitude, as reported in the Section 2 and Section 3. Corner and edge singularities were identified and excluded from interpretation, and model-dependent nonrepresentative peaks were treated accordingly. The spatial distribution and magnitude of stresses aligned with recent FEA reports, supporting the numerical plausibility of the models. Because all configurations were analyzed under identical materials, boundary conditions, and contact definitions, like-for-like comparison yields high internal validity for ranking their mechanical behavior. The absence of direct experimental validation, therefore, affects only the absolute values and not the comparative interpretation of the results.

### 4.1. Central Findings

#### 4.1.1. Oblique Loading Dominates the Response

The loading direction plays an important role in the biomechanical behavior: oblique test condition (300 N at 30°) results in substantially higher stresses compared to axial (400 N), particularly at implant neck/first thread and in the zones close to the crown’s antirotation element/screw-access channel. At the implant level, TRI Matrix P37 showed the highest peaks under oblique loading (e.g., P37-S ≈ 2493.3 MPa at neck/first thread), compared to TRI Matrix P45 and TRI Octa. At the crown level, TRI Matrix P45-L displayed the highest stress concentration within the antirotation zone alone (≈1109.8 MPa axial; ≈1462.4 MPa oblique) (Table 6 and Figure A3). Although high stresses (σ_vM_ > σ_adm_) exist in this region, the remaining zirconia volume stays in a comfortable static safety margin. In clinical practice, this suggests that static fracture is unlikely in properly functioning restoration, but fatigue-related damage becomes relevant when oblique/eccentric loading is present (Table 6 and Figure A3). These results are consistent with recent FEA literature [12,16,17,25,65,66]. To complement the qualitative interpretation of loading-direction effects, we also examined the stress ratios within our models when switching from axial (400 N) to oblique loading (300 N at 30°). Across all configurations, oblique loading increased von Mises stresses at the implant neck by roughly 1.7–2.0× (Table 5). In the 3Y-TZP crown, the corresponding rise in σ_1_ and σ_vM_ was 1.3–1.4× (Table 6). Direct numerical comparison between different FEA studies is not methodologically appropriate because of the substantial variation in geometries and load protocols, but several works report the same directional effect: higher stresses under oblique loading, with concentration at the implant neck and crestal bone [67], greater component deformation and micromotion [68], and consistently elevated stresses in implant–abutment assemblies under nonaxial forces [12]. The fact that our stress ratios fall within the range of these published patterns supports the biomechanical consistency of the present findings.

#### 4.1.2. The Composite Does Not Carry Any Substantial Load

Occlusal forces bypass the composite and are transferred to the much stiffer crown and metallic components. Screw preload does not induce significant stresses in this volume (Figure 7). Clinically, this indicates that the composite is not a stress-critical element in the studied cases.

#### 4.1.3. TRI Matrix P37 Versus P45 Has Different Load Transfer Architectures

Preload (Step 1) forms a compression cone beneath the screw head; this stiffened volume attracts additional load in Step 2. TRI Matrix P37′s broader cone base results in a wider platform-level load distribution and a more uniform transmission of forces in the crown. In TRI Matrix P45, most of the load is concentrated under the screw head, which reduces the contribution of the platform and increases local axial compression. The effect is stronger with the long screw, which creates a larger stiffened volume, particularly for TRI Matrix P45. In TRI Matrix P37, on the other hand, the short screw is optimal for achieving a balanced load distribution at the implant platform level. These findings are consistent with how preload and interface geometry determine load paths in screw-retained implant restorations [16,17]. Also, for TRI Matrix P37, at the implant neck/first thread the stress regime is mostly compressive and the fracture risk under static conditions is low. However, compression-led fatigue/fretting can, nonetheless, be expected in repeated oblique loading, which accentuates the need for clinical management of parafunctions and occlusal stabilization.

#### 4.1.4. Antirotation Elements Are Local Stress Raisers

Peaks in the antirotation recess (especially in the crown) and at the implant neck/first thread are positioned where abrupt changes in cross-sections occur. Without filleting, sharp edges generate classic FEA numerical peaks. Their point-like occurrence on high-magnification maps (Figure A3) and sensitivity to contact and meshing warrant reading them as model-dependent singularities instead of a sign of volumetric failure [25,65]. At platform level, distributions can also become irregular when antirotation edges are misaligned with the oblique loading vector. Prosthetic planning should consider indexing strategies that avoid direct alignment between antirotation edges and the dominant load directions.

### 4.2. Comparative Appraisal of Configurations

#### 4.2.1. Implants (Ti-6Al-4V)

Under oblique loading (300 N at 30°), TRI Matrix P37 consistently showed the highest von Mises stress at the implant neck and first thread. Lower peak values of TRI Matrix P45 and TRI Octa suggest that a good amount of preload-induced compression concentrates under the screw head (TRI Matrix P45) or spreads more evenly over the abutment–implant interface (TRI Octa). Because the analysis is quasi-static, these cervical peaks in P37 are best interpreted as potential fatigue-relevant hotspots under repeated oblique masticatory loading, rather than as predictors of immediate static failure. In axial loading, values decrease considerably relative to oblique loading. During preload, stress variations were present but were smaller than under oblique loading, which is consistent with how pretightening creates a compressive cone that prepares the load path without, by itself, creating the critical scenario [16,17]. The maxima under oblique loading in TRI Octa were inside the internal recess and were mostly compressive. Minimal internal filleting and improved surface finish in this area would reduce these values without affecting abutment-mediated transfer.

#### 4.2.2. 3Y-TZP Crown

For TRI Matrix P45-L and P45-S, the highest tensile σ_1_ values are restricted to the antirotation recess, where the reduced radius and unfavorable contact conditions generate a highly uneven stress distribution (Figure 9 and Figure A2). This more concentrated load transfer in P45 justifies the σ_1_ hotspot observed in the screw access/antirotation zone [16]. By comparison, TRI Octa exhibited lower σ_1_ values and more diffuse distribution, consistent with a less-focused load path in the crown. Table 6 is retained only as a qualitative descriptor and is not used to infer ceramic failure risk.

#### 4.2.3. Abutment (Ti-Base, TRI Octa)

Local stress peaks appeared at sharp edges and corners and were point-like and of small volume. These are influenced by contact definition and mesh discretization and, clinically, tend to be attenuated by superficial plasticity or chamfering resulting from manufacturing and finishing. Abutment-type comparisons similarly indicate that Ti-base-supported hybrid designs can move part of the burden to the Ti-base, while retention screw and/or implant collar may become the limiting regions depending on the configurations [27]. The more practical consequence is the need to refine microfillets and control surface quality, rather than to reconsider the overall connection concept [16].

### 4.3. Mechanical Interpretation

The results can be explained by the mechanics of bolted joints. In Step 1 (preload), a conical compressive zone forms beneath the screw head. In Step 2 (occlusal loading), the interface design controls the load transfer path: TRI Matrix P37 promotes platform-level sharing, while TRI Matrix P45 concentrates stresses beneath the screw head.

Screw length modulates this effect. A long screw enlarges the stiffened zone (more pronounced in TRI Matrix P45), while in TRI Matrix P37 a short screw further encourages platform-level sharing.

An increased ceramic volume beneath the screw head (with a longer screw) does not automatically increase the strength of the restoration. The compression cone stiffens that volume and attracts load, creating local peaks at the antirotation feature. Mechanical optimization should balance screw length, screw head geometry, and crown blending. Peaks at antirotation features edges are stress raisers and must be treated with caution, as they are sensitive to meshing and contact modeling. All of the above aligns with recent FEA reports on the role of nonaxial loading and preload in stress distribution [16,17,25,65].

### 4.4. Clinical Implications

#### 4.4.1. A Cautious Clinical Interpretation

Oblique forces mainly induce overloading in the tested assemblies, with maxima at the implant neck and first thread and near crown antirotation recesses. Purely axial loading is, in general, more favorable. When the occlusal scheme is predominantly axial, TRI Matrix P37 will allow for more uniform stress sharing at implant platform level. In the presence of oblique or complex load components, TRI Matrix P45 is mechanically robust, but caution is required with high-stress areas at the crown level, especially with longer screws. TRI Octa is robust under axial and preload conditions, with small-volume peaks under oblique loading, which is compatible with healthy clinical stability. This agrees with FEA evidence on sensitivity to nonaxial components and with clinical evidence that supports screw-retained solutions for maintenance and for managing complications. Local overloading can be mitigated by inserting the implant in an appropriate (prosthetically driven) position relative to the resultant occlusal vector. This underlines the importance and need for digital presurgical and preprosthetic planning, including occlusal and parafunctional examination [12,16,17,25,65,69].

#### 4.4.2. Abutment-Free Configurations (TRI Matrix), Potential Clinical Advantages

Mechanical. Reducing the number of interfaces will reduce cumulative tolerances and contact surfaces relaxation under loading. The literature indicates that internal contact geometry affects preload loss and loosening risk, which means that eliminating an interface can be favorable, although system-specific validation is needed [16,17,70].

Microleakage. Microgaps at implant–abutment junctions are well documented in vitro. Precision of fit and the number of interfaces are responsible for bacterial colonization, supporting the theoretical proposition “fewer interfaces equals fewer microspaces and less bacterial leakage” [3,71].

Biologic (soft-tissue stability). The “One abutment, one time” concept has been linked with healthy soft-tissue dynamics and added marginal stability. This conceptual foundation can be translated to abutment-free configuration by means of fewer transmucosal interface manipulations [69,72,73,74,75].

Cement-related risk control. In some recent studies, screw-retained options were shown to reduce the risk for biological complications related to intraoral cementation. Other syntheses document no consistent differences between retention forms, calling for caution and individualized treatment planning [74].

Aesthetics/function. Angled screw channels allow relocation of the access hole to occlusal/palatal zones, preserving the aesthetics without the need for angulated abutments or cementation [76,77,78,79].

#### 4.4.3. Abutment Type and Material Pairing

Clinically, the differences observed between the Ti-base assembly (TRI Octa) and the abutment-free, direct-to-implant concept (TRI Matrix) can be interpreted through the combined effect of interface architecture and material stiffness. Ti-base restorations include a ductile titanium component that can redistribute local stresses and confine critical peaks to small internal regions of the connection, as suggested by our Octa maps. Conversely, the abutment-free zirconia crown—screw-retained directly to the implant—eliminates the intermediate interface but increases the rigidity of the crown–implant coupling, which may concentrate stresses around the screw-access and antirotation features under nonaxial loading. These differences are consistent with experimental and numerical data on abutment-free zirconia interfaces and hybrid Ti-base assemblies [1,29,30]. These differences suggest that the abutment-free configuration may favor prosthetic integrity in well-aligned loading scenarios, while Ti-base assemblies could remain advantageous under off-axis or fatigue loading conditions.

### 4.5. Study Limitations

#### 4.5.1. Material and Geometry Assumptions

All materials were modeled as homogeneous, isotropic, and linear elastic (ν = 0.30). Threads were not explicitly modeled (component-level assessment), and crown geometry was simplified and axially symmetric. Possible effects related to microstructure or detailed thread geometry may, therefore, be attenuated in global stress outcomes.

#### 4.5.2. Contact Conditions

Critical interfaces were modeled as nonlinear frictional contact (μ = 0.10), with an Augmented Lagrange algorithm and small-sliding formulation. In the TRI Matrix assembly, potential initial microclearance at one interface could alter the hierarchy of contact engagement and the load path. The adopted μ represents a relatively low-friction condition consistent with commonly used implant FEA assumptions and a controlled contact definition. Clinically, higher friction (μ ≈ 0.2–0.3) may occur under drier tightening or different surface conditions, which, under torque-controlled tightening, would be expected to reduce the achieved preload for the same applied torque and potentially increase interfacial micromobility, particularly relevant for abutment-free connections. In the present simulations, preload was imposed as a bolt pretension (812 N), so μ mainly influences interfacial stick–slip and shear transfer (and, thus, local stress peaks), rather than the magnitude of the clamping force. As the same μ was applied across all configurations, comparative differences are expected to remain similar; however, without a dedicated sensitivity analysis, changes in absolute peak stresses or hotspot locations cannot be excluded, so maximum values should be interpreted cautiously.

#### 4.5.3. Screw Preload

Preload was applied as a bolt load equivalent to T = 35 N·cm (F = 812 N) in Step 1, with Step 2 loads superimposed on the preloaded state. Because the retention screw was represented as a smooth body (threads not explicitly modeled), stress concentrations at the first engaged thread and the detailed preload distribution along the thread engagement may be underestimated compared with a fully threaded model. Variability in torque coefficient (K), friction beneath the screw head, and lubrication was not explored parametrically, which means that results reflect a single pretightening condition.

#### 4.5.4. Occlusal Loading Scheme

We applied 400 N axial and 300 N at 30° (buccolingual plane) as a uniform remote force over a finite patch (5 mm^2^) to avoid singularities. Real multicontact and eccentric occlusion can channel forces along different paths to this standardized patch [80].

#### 4.5.5. Boundary Conditions and Edge Effects

Fixing the cut faces can over-stiffen the bone block and induce edge effects; therefore, we restricted interpretation to functional interfaces. Although displacement fields were computed, absolute deformations depend strongly on the imposed constraints and bolt preload implementation, so deformation was not used as a primary endpoint and the bone block served only as compliant support.

#### 4.5.6. Meshing and Convergence Verification

SOLID187 elements with local refinements were used. Convergence was confirmed by successive refinements in regions of interest. Point-like peaks remain sensitive to mesh density and contact settings, and corner fillets were not calibrated to manufacturing tolerances. This means that some numerical values reflect modeling and meshing idealizations.

#### 4.5.7. Post-Processing and Display Thresholds

Stress maps use predefined display windows and banding, which can visually compress or saturate gradients. For this reason, maps and tabulated values were interpreted together, focusing on consistent patterns across configurations.

#### 4.5.8. Bone Modeling

Bone was modeled as a finite-stiffness support without remodeling [53]. Because cortical and trabecular bone were modeled as homogeneous and isotropic, the stresses predicted at the implant neck may not fully reflect the behavior of real cortical bone, which is structurally directional. Under oblique loading, bending forces accentuate these local stiffness variations, which can affect both the magnitude and the spatial extent of the cortical hotspot at the implant neck/cortical interface. Even so, all configurations in this study were analyzed using the same material assumptions, so any deviation in absolute values would affect them in the same way. For this reason, the simplification does not alter the comparative interpretation of the results or the relative differences observed between the Matrix and Octa assemblies. However, the model should not be used to infer bone-strain thresholds, remodeling, or marginal bone-loss predictions.

#### 4.5.9. Analysis Regime

The analysis is quasi-static. Fatigue and cumulative damage processes were not modeled. These findings should not be interpreted as predictions of fatigue life or fracture probability, but, rather, as a comparative indication of stress transfer within and between the investigated assemblies.

#### 4.5.10. Unexplored Sensitivities

No systematic sensitivity analysis was performed for friction coefficient and contact conditions, possible microclearances and misalignments, orientation of antirotation features relative to oblique load vectors, occlusal patch topology and size, realistic multicontact occlusion patterns, torque variability, surface finish (chamfers and microfillets), or explicit thread modeling. All may influence the location and amplitude of local peaks, although the global comparative trends are likely to be preserved.

### 4.6. Perspectives and Future Research

#### 4.6.1. ISO-Style Fatigue Testing

A next step is ISO-14801-style fatigue testing to correlate Step 2 peaks with fatigue strength, using S–N curves for Ti-6Al-4V and sizing criteria (e.g., Goodman/Soderberg) [41].

#### 4.6.2. Validation

- In vitro (photoelasticity/DIC) focused on implant neck and antirotation, to provide full field stress and strain patterns for qualitative concordance with FEA hotspots.
- In vivo focused on monitoring removal torque values and prosthetic complications for P37/P45/Octa.

#### 4.6.3. Damage Maps and Fretting-Fatigue

Goodman/Soderberg-based damage mapping and fretting–fatigue assessment at the neck and first thread in oblique loading scenarios would help quantify lifetime-related risks.

#### 4.6.4. Ceramic-Specific Modeling

For 3Y-TZP, probabilistic σ_1_-based approaches (Weibull/defect-volume) and slow-crack-growth modeling in moist environments, aligned with ISO 6872:2024 for dental ceramics, could turn local peaks into volume-based risk estimates [81].

#### 4.6.5. Sensitivity and Uncertainty in Contact and Preload

Targeted sensitivity runs for μ, microclearance, and preload scatter would quantify robustness of the comparative conclusions [82,83].

#### 4.6.6. Length Series for Screws

Extending screw-length series could clarify the balance between platform-level sharing and concentrations beneath the screw head.

#### 4.6.7. Indexing Orientation

Varying the antirotation orientation relative to the oblique load vector could identify orientation-dependent effects in the crown and implant neck.

#### 4.6.8. Multipoint Occlusion from Clinical Measurements

Translating to FEA measured occlusal contacts (e.g., T-Scan: amplitudes/timing, parafunctions) could enable individualized scenarios and “in vivo-like” validation of distributions [84].

#### 4.6.9. Fillet Optimization

Multiobjective optimization of antirotation fillet radius (crown/implant) versus local peaks, constrained by contact behavior and screw-channel position, could identify safer geometries.

#### 4.6.10. Abutment-Free (TRI Matrix)

Eliminating the implant–abutment interface reduces potential microleakage/biofilm sites and associated biomechanical changes reported across connection types, while preventing titanium “shine-through” at soft-tissue margin. Dedicated clinical series are required to convert this potential into direct clinical evidence [85,86,87,88,89].

## 5. Conclusions

This 3D FEA compared a conventional tissue-level implant with an engaging Ti-base (TRI Octa) against abutment-free configurations (TRI Matrix, platforms P37/P45, short and long retention screw), under a standardized screw preload (35 N·cm) and axial and oblique occlusal loads. Within the model’s assumptions, oblique components governed the mechanical response: a 300 N force at 30° systematically amplified stresses relative to a 400 N axial load and produced peaks at the implant neck and first thread and at the crown’s screw-access and antirotation features.

At the implant level, TRI Matrix P37 was the critical scenario under oblique loading, whereas TRI Matrix P45 remained comparable to TRI Octa, or slightly below. In TRI Octa, peaks were small in volume and located in the implant’s internal recess, showing that eliminating the Ti-base/abutment shifts the dominant implant hotspot toward the neck/first-thread region under the same loading.

In the 3Y-TZP crown, maxima were strictly local around antirotation features. The highest values occurred in TRI Matrix P45-L, while TRI Matrix P37-L was close to control. Taken together, increasing platform width from P37 to P45 reduces implant-neck peaks toward the TRI Octa control, but increases strictly local crown stresses at the antirotation/screw-access region. Screw preload (35 N·cm → 812 N bolt load) and screw length modulated the transfer path: TRI Matrix P37 favored implant platform-level sharing (especially with a short screw), while in P45, compression concentrated beneath the screw head (more so with a long screw), with long screws also accentuating the local crown peak adjacent to the screw-access/antirotation features.

The composite volume did not act as a significant load-bearing element, and bone remote from interfaces stayed below 30 MPa, supporting a focus on prosthetic components and interfaces.

Clinically, in occlusal schemes with predominantly axial loads, TRI Matrix P37 supports more uniform implant platform-level distribution (with lower demands on the crown), while in the presence of oblique/eccentric components, TRI Matrix P45 offers static robustness but needs attention to crown-level high-stress regions, especially with long screws. TRI Octa remains robust with small-volume peaks even under oblique loading. Future work should incorporate ISO-style fatigue testing, sensitivity analyses for friction and preload, evaluation of antirotation element indexing orientation, and experimental and clinical validation to convert these biomechanical inferences into recommendations supported by robust clinical evidence.

## Figures and Tables

**Table 1:** OEM (SKU) identifiers of components (including the control configuration).

Configuration	Implant (OEM/SKU)	Prosthetic Platform (mm)	Ti-Base (OEM/SKU)	Clinical Screw (OEM/SKU)
TRI Octa + Ti-base + ZrO_2_ 3Y-TZP crown (control)	TO41M10	4.8	TO70-07-F	RS-TO
TRI Matrix P37 + ZrO_2_ 3Y-TZP crown + short screw	TLM-37-10-P45	3.7	-	M-SCRW-2.8
TRI Matrix P37 + ZrO_2_ 3Y-TZP crown + long screw	TLM-37-10-P45	3.7	-	M-SCRW-2.8-L
TRI Matrix P45 + ZrO_2_ 3Y-TZP crown + short screw	TLM-41-10-P45	4.5	-	M-SCRW-2.8
TRI Matrix P45 + ZrO_2_ 3Y-TZP crown + long screw	TLM-41-10-P45	4.5	-	M-SCRW-2.8-L

**Table 2:** Material properties considered in the analysis.

Material	E (GPa)	ν	σ_y_ (MPa)	σ_u_ (MPa)	Source
Cortical bone	15	0.3	-	-	[50,51,53,54,55]
Trabecular bone	7		-	-	[50,51,54]
Crown (ZrO_2_ 3Y-TZP)	200		1100	1500	[46,48]
Implant (Ti-6Al-4V)	110		800	1000	[47]
Ti-base (Ti-6Al-4V)	110		800	1000	[47]
Crown retention screw (Ti-6Al-4V)	110		800	1000	[47]
Composite	10		-	-	[49]

**Table 3:** Contact definitions used in the five configurations (Ansys Mechanical).

Contact Type	Configuration	Contact Pair	Formulation/Notes
Frictional (μ = 0.10)	TRI Matrix	Crown–implant	Nonlinear surface-to-surface; separation + sliding allowed; active simultaneously with crown–screw (no exclusivity).
Frictional (μ = 0.10)	TRI Matrix	Retention screw–crown	Nonlinear surface-to-surface; separation + sliding allowed.
Frictional (μ = 0.10)	TRI Octa	Implant–Ti-base	Nonlinear surface-to-surface; MPC applied on antirotation features (see text below).
Frictional (μ = 0.10)	TRI Octa	Retention screw–Ti-base	Nonlinear surface-to-surface; separation + sliding allowed.
Bonded	All	Crown–composite	Perfectly tied (no slip/separation).
Bonded	TRI Octa	Crown–Ti-base	Perfectly tied (cemented interface).
Bonded	All	Implant–cortical/trabecular bone	Perfectly tied (osseointegration assumed).
Bonded	All	Cortical–trabecular bone	Perfectly tied (tissue continuity).

**Table 4:** Discretization statistics (final meshes, SOLID187, global element size 0.5 mm, local refinements 0.06–0.2 mm).

Configuration	Elements	Nodes
TRI^®^ Matrix P37-S	376,140	594,388
TRI^®^ Matrix P37-L	376,713	594,003
TRI^®^ Matrix P45-S	400,092	634,325
TRI^®^ Matrix P45-L	399,967	633,069
TRI^®^ Octa (Ti-Base Engaging)	698,101	1,155,110

**Table 5:** Maxima of von Mises stress (σ_max_ (MPa)) in the implant (Ti-6Al-4V) for screw preload, axial 400 N, and oblique 300 N at 30°. Reported: σ_max_, σ_max_/σ_adm_, Δ% vs. TRI Octa, σ_max_/σ_y_ and location of maxima. Δ% vs. TRI Octa is shown for quick reference only and is not used for between-configuration comparison.

Configuration	Load	σ_max_ (σ_vM_) (MPa)	σ_max_/σ_adm_ (−)	Δ% vs. TRI Octa (%)	σ_max_/σ_y_ (−)	Location of Maximum
TRI Octa	Preload	602.0	1.20	0.0	0.75	Internal relief (local stress concentrator).
TRI Matrix P37-S	Preload	1086.0	2.17	80.4	1.36	Implant neck/first thread (at the interface with cortical bone).
TRI Matrix P37-L	Preload	1133.0	2.27	88.2	1.42	Implant neck/first thread (at the interface with cortical bone).
TRI Matrix P45-S	Preload	665.0	1.33	10.5	0.83	Implant neck/first thread (at the interface with cortical bone).
TRI Matrix P45-L	Preload	665.0	1.33	10.5	0.83	Implant neck/first thread (at the interface with cortical bone).
TRI Octa	Axial 400 N	745.0	1.49	0.0	0.93	Internal relief (local stress concentrator).
TRI Matrix P37-S	Axial 400 N	1393.0	2.79	87.0	1.74	Implant neck/first thread (at the interface with cortical bone).
TRI Matrix P37-L	Axial 400 N	1148.0	2.30	54.1	1.44	Implant neck/first thread (at the interface with cortical bone).
TRI Matrix P45-S	Axial 400 N	744.0	1.49	−0.1	0.93	Implant neck/first thread (at the interface with cortical bone).
TRI Matrix P45-L	Axial 400 N	803.0	1.61	7.8	1.00	Implant neck/first thread (at the interface with cortical bone).
TRI Octa	Oblique 300 N at 30°	1124.0	2.25	0.0	1.41	Internal relief (local; small volume; compression-dominant).
TRI Matrix P37-S	Oblique 300 N at 30°	2493.3	4.99	121.8	3.12	Implant neck/first thread (at the interface with cortical bone).
TRI Matrix P37-L	Oblique 300 N at 30°	1959.0	3.92	74.3	2.45	Implant neck/first thread (at the interface with cortical bone).
TRI Matrix P45-S	Oblique 300 N at 30°	1056.0	2.11	−6.0	1.32	Implant neck/first thread (at the interface with cortical bone).
TRI Matrix P45-L	Oblique 300 N at 30°	1129.0	2.26	0.4	1.41	Implant neck/first thread (at the interface with cortical bone).

**Note:** σ_adm_ (metallic alloy) = 500 MPa (σ_y_ ≈ 800 MPa; SF = 1.6). Δ% vs. TRI Octa is calculated for the same loading type. σ_max_/σ_y_ computed with σ_y_ = 800 MPa. Conventions for “Location”: TRI Matrix—implant neck/first thread (at the interface with cortical bone); TRI Octa—Ti-base octagonal antirotation interface (local stress concentrator; small volume; predominantly compressive regime). In cases where σ_max_ exceeds ~2000 MPa, the peak is a mesh-dependent singular spike at a sharp geometric stress raiser, confined to a negligible vicinity; it is reported only to flag hotspot location and should not be interpreted as immediate static failure. Values are rounded to one decimal place (MPa and Δ%).

**Table 6:** Crown (3Y-TZP) von Mises maxima (σ_max_ (MPa)) for screw preload, axial 400 N, and oblique 300 N at 30°. Reported: σ_max_, Δ% vs. TRI Octa, and location of maxima. Δ% values are provided for quick reference only. Comparative interpretation relies on stress-field distributions.

Configuration	Load	σ_max_ (σ_vM_) (MPa)	Δ% vs. TRI Octa (%)	Location of Maximum
TRI Octa	Preload	332.0	0.0	Antirotation feature/screw-access (localized peak).
TRI Matrix P37-S	Preload	370.0	11.4	Antirotation feature/screw-access (localized peak).
TRI Matrix P37-L	Preload	428.0	28.9	Antirotation feature/screw-access (localized peak).
TRI Matrix P45-S	Preload	659.0	98.5	Antirotation feature/screw-access (localized peak).
TRI Matrix P45-L	Preload	773.0	132.8	Antirotation feature/screw-access (localized peak).
TRI Octa	Axial 400 N	416.0	0.0	Antirotation feature/screw-access (localized peak).
TRI Matrix P37-S	Axial 400 N	397.0	−4.6	Antirotation feature/screw-access (localized peak).
TRI Matrix P37-L	Axial 400 N	384.0	−7.7	Antirotation feature/screw-access (localized peak).
TRI Matrix P45-S	Axial 400 N	1005.0	141.6	Antirotation feature/screw-access (localized peak).
TRI Matrix P45-L	Axial 400 N	1110.0	166.8	Antirotation feature/screw-access (localized peak).
TRI Octa	Oblique 300 N at 30°	442.0	0.0	Antirotation feature/screw-access (localized peak).
TRI Matrix P37-S	Oblique 300 N at 30°	525.0	18.8	Antirotation feature/screw-access (localized peak).
TRI Matrix P37-L	Oblique 300 N at 30°	457.0	3.4	Antirotation feature/screw-access (localized peak).
TRI Matrix P45-S	Oblique 300 N at 30°	1024.0	131.7	Antirotation feature/screw-access (localized peak).
TRI Matrix P45-L	Oblique 300 N at 30°	1462.0	230.8	Antirotation feature/screw-access (localized peak).

**Note:** For 3Y-TZP, interpretation is based on σ_1_ (primary metric); σ_vM_ is provided as a complementary descriptor. Convention for “Location”: antirotation feature/screw-access (localized peak). Units in header (MPa); rounding is to one decimal place (MPa and Δ%).

**Table 7:** Ti-base (TRI Octa). Preload, axial 400 N, oblique 300 N at 30°.

Load	σ_max_ (σ_vM_) (MPa)	σ_max_/σ_adm_ (−)	σ_max_/σ_y_ (−)	Location of Maximum
Preload	742.0	1.48	0.93	Edges/corners, small volume.
Axial 400 N	748.0	1.50	0.94	Edges/corners, small volume.
Oblique 300 N at 30°	1221.0	2.44	1.53	Edges/corners, small volume.

**Note:** Metallic component (Ti-6Al-4V), von Mises criterion; σ_adm_ = 500 MPa (σ_y_ ≈ 800 MPa; SF = 1.6); σ_max_/σ_y_ with σ_y_ = 800 MPa. Values represent σ_vM_, _max_ on the Ti-base (TRI Octa) for the three scenarios; maxima are local (edges/corners, small volume). Values are reported to indicate hotspot location and are not used to compare across configurations.

## Data Availability

The original contributions presented in the study are included in the article, further inquiries can be directed to the corresponding authors.

## Abbreviations

The following abbreviations are used in this manuscript:

3D	Three-dimensional
FEA	Finite Element Analysis
CAD	Computer-aided design
ISO	International Organization for Standardization
SI	International System of Units
DIC	Digital image correlation
MPC	Multipoint constraint
OEM	Original equipment manufacturer
SKU	Stock keeping unit
SF	Safety factor
STEP	Standard for the Exchange of Product model data
SOLID187	10-node structural solid finite element
3Y-TZP	3 mol% yttria-stabilized tetragonal zirconia polycrystal
Ti-6Al-4V	Ti alloy, grade 5
Ti-base	Titanium-base abutment
T-Scan	Computerized occlusal analysis system
E	Young’s modulus (GPa)
ν	Poisson’s ratio
σ_y_	Yield strength (MPa)
σ_u_	Ultimate strength (flexural for zirconia) (MPa)
σ_3_	Minimum principal stress
σ_1_	Maximum principal stress
μ	Coefficient of friction
σ_vM_	von Mises equivalent stress
σ_adm_	Admissible stress

## References

[1] Mugri M.H., Reddy N.K., Sayed M.E., Mattoo K., Qomari O.M., Alnaji M.M., Mshari W.A., Alqarawi F.K., AlResayes S.S., Alshaibani R.M. (2025). Stress Assessment of Abutment-Free and Three Implant–Abutment Connections Utilizing Various Abutment Materials: A 3D Finite Element Study of Static and Cyclic Static Loading Conditions. J. Funct. Biomater..

[2] Kim J.-H., Noh G., Hong S.-J., Lee H. (2020). Biomechanical Stress and Microgap Analysis of Bone-Level and Tissue-Level Implant Abutment Structure According to the Five Different Directions of Occlusal Loads. J. Adv. Prosthodont..

[3] Mao Z., Beuer F., Wu D., Zhu Q., Yassine J., Schwitalla A., Schmidt F. (2023). Microleakage along the Implant–Abutment Interface: A Systematic Review and Meta-Analysis of in Vitro Studies. Int. J. Implant Dent..

[4] Wittneben J.-G., Joda T., Weber H.-P., Brägger U. (2017). Screw Retained vs. Cement Retained Implant-Supported Fixed Dental Prosthesis. Periodontol. 2000.

[5] Lee H., Jo M., Sailer I., Noh G. (2022). Effects of Implant Diameter, Implant-Abutment Connection Type, and Bone Density on the Biomechanical Stability of Implant Components and Bone: A Finite Element Analysis Study. J. Prosthet. Dent..

[6] Lago L., da Silva L., Fernández-Formoso N., Rilo B. (2023). Systematic Assessment of Soft Tissue Level and Bone Level Dental Implants. J. Oral Maxillofac. Surg. Med. Pathol..

[7] Araki H., Nakano T., Ono S., Yatani H. (2020). Three-Dimensional Finite Element Analysis of Extra Short Implants Focusing on Implant Designs and Materials. Int. J. Implant Dent..

[8] Mosavar A., Nili M., Hashemi S.R., Kadkhodaei M. (2017). A Comparative Analysis on Two Types of Oral Implants, Bone-Level and Tissue-Level, with Different Cantilever Lengths of Fixed Prosthesis. J. Prosthodont..

[9] Lee J.-H., Jang H.Y., Lee S.Y. (2021). Finite Element Analysis of Dental Implants with Zirconia Crown Restorations: Conventional Cement-Retained vs. Cementless Screw-Retained. Materials.

[10] Avağ C., Akkocaoğlu M. (2023). The Evaluation of Stress on Bone Level and Tissue Level Short Implants: A Finite Element Analysis (FEA) Study. J. Stomatol. Oral Maxillofac. Surg..

[11] Mitra D., Gurav P., Rodrigues S., Khobragade B., Mahajan A. (2023). Evaluation of Stress Distribution in and around Dental Implants Using Three Different Implant-Abutment Interfaces with Platform-Switched and Non-Platform-Switched Abutments: A Three-Dimensional Finite Element Analysis. J. Dent. Res. Dent. Clin. Dent. Prospect..

[12] Yadav K., Kumar S., Aggarwal R., Kaur I., Goyal A., Sharma R., Banjara S. (2025). Finite Element Analysis of Platform Switching Effects on Stress Distribution in Posterior Implants Placed in Different Bone Types Under Axial and Oblique Loading Conditions. Cureus.

[13] Rasouli-Ghahroudi A.A., Geramy A., Yaghobee S., Khorsand A., Yousefifakhr H., Rokn A., Soolari A. (2015). Evaluation of Platform Switching on Crestal Bone Stress in Tapered and Cylindrical Implants: A Finite Element Analysis. J. Int. Acad. Periodontol..

[14] Afazal M., Gupta S., Tevatia A., Afreen S., Chanda A. (2023). Computational Investigation of Dental Implant Restoration Using Platform-Switched and -Matched Configurations. Computation.

[15] Aslam A., Hassan S.H., Aslam H.M., Khan D.A. (2019). Effect of Platform Switching on Peri-Implant Bone: A 3D Finite Element Analysis. J. Prosthet. Dent..

[16] Al-Sanea A., Aktas S., Celik T., Kisioglu Y. (2023). Effects of the Internal Contact Surfaces of Dental Implants on Screw Loosening: A 3-Dimensional Finite Element Analysis. J. Prosthet. Dent..

[17] Szajek K., Wierszycki M. (2023). Screw Preload Loss under Occlusal Load as a Predictor of Loosening Risk in Varying Dental Implant Designs. J. Mech. Behav. Biomed. Mater..

[18] Sagheb K., Görgen C.-I., Döll S., Schmidtmann I., Wentaschek S. (2023). Preload and Friction in an Implant–Abutment–Screw Complex Including a Carbon-Coated Titanium Alloy Abutment Screw: An in Vitro Study. Int. J. Implant Dent..

[19] Sun Y., Shukla A., Ramachandran R.A., Kanniyappan H., Yang B., Harlow R., Campbell S.D., Thalji G., Mathew M. (2024). Fretting-Corrosion at the Implant-Abutment Interface Simulating Clinically Relevant Conditions. Dent. Mater..

[20] Anniwaer A., Yin Z., Zhu J., Jin C., Muhetaer A., Huang C. (2025). Comparison of Three Implant Systems under Preload Loss: A Finite Element Analysis Validated by Digital Image Correlation Methods. J. Prosthodont. Res..

[21] de Holanda Cavalcanti Pereira A.K., de Oliveira Limirio J.P.J., Cavalcanti do Egito Vasconcelos B., Pellizzer E.P., Dantas de Moraes S.L. (2024). Mechanical Behavior of Titanium and Zirconia Abutments at the Implant-Abutment Interface: A Systematic Review. J. Prosthet. Dent..

[22] Deste Gökay G., Oyar P., Gökçimen G., Durkan R. (2024). Static and Dynamic Stress Analysis of Different Crown Materials on a Titanium Base Abutment in an Implant-Supported Single Crown: A 3D Finite Element Analysis. BMC Oral Health.

[23] Poovarodom P., Rungsiyakull C., Suriyawanakul J., Li Q., Sasaki K., Yoda N., Rungsiyakull P. (2023). Effect of Gingival Height of a Titanium Base on the Biomechanical Behavior of 2-Piece Custom Implant Abutments: A 3-Dimensional Nonlinear Finite Element Study. J. Prosthet. Dent..

[24] Falcinelli C., Valente F., Vasta M., Traini T. (2023). Finite Element Analysis in Implant Dentistry: State of the Art and Future Directions. Dent. Mater..

[25] Yang B., Landa A.I., Heuberger P., Ploeg H.-L. (2024). Effects of Dental Implant Diameter and Tapered Body Design on Stress Distribution in Rigid Polyurethane Foam during Insertion. Med. Eng. Phys..

[26] Tribst J.P.M., Özkara N., Blom E.J., Kleverlaan C.J., Ausiello P., Bruhnke M., Feilzer A.J., Dal Piva A.M.d.O. (2025). The Effect of Implant–Abutment Contact Area on the Stress Generation of Bone-Level and Tissue-Level Implants. Appl. Sci..

[27] Pumnil S., Rungsiyakull P., Rungsiyakull C., Elsaka S. (2022). Effect of Different Customized Abutment Types on Stress Distribution in Implant-Supported Single Crown: A 3D Finite Element Analysis. J. Prosthodont..

[28] TRI® Dental Implants Int. AG. MATRIX Line. Hünenberg, Switzerland. https://tri-implants.swiss/en/matrix-line/.

[29] Südbeck S., Buser R., Reymus M., Hoffmann M., Edelhoff D., Stawarczyk B. (2023). A New Implant System with Directly Screwed Supraconstructions: Impact of Restoration Material and Artificial Aging on the Bending Moment. Int. J. Prosthodont..

[30] Hjerppe J., Jung R.E., Hämmerle C.H., Özcan M., Mühlemann S. (2022). Mechanical Stability of Fully Personalized, Abutment-Free Zirconia Implant Crowns on a Novel Implant-Crown Interface. J. Dent..

[31] Brizuela-Velasco A., Pérez-Pevida E., Jiménez-Garrudo A., Gil-Mur F.J., Manero J.M., Punset-Fuste M., Chávarri-Prado D., Diéguez-Pereira M., Monticelli F. (2017). Mechanical Characterisation and Biomechanical and Biological Behaviours of Ti-Zr Binary-Alloy Dental Implants. BioMed Res. Int..

[32] Shemtov-Yona K., Rittel D. (2015). On the Mechanical Integrity of Retrieved Dental Implants. J. Mech. Behav. Biomed. Mater..

[33] Kihara H., Hatakeyama W., Kondo H., Yamamori T., Baba K. (2022). Current Complications and Issues of Implant Superstructure. J. Oral Sci..

[34] Osman R.B., Swain M.V. (2015). A Critical Review of Dental Implant Materials with an Emphasis on Titanium versus Zirconia. Materials.

[35] Kaleli N., Sarac D., Külünk S., Öztürk Ö. (2018). Effect of Different Restorative Crown and Customized Abutment Materials on Stress Distribution in Single Implants and Peripheral Bone: A Three-Dimensional Finite Element Analysis Study. J. Prosthet. Dent..

[36] Chang C.-L., Karmakar R., Mukundan A., Lu S.-H., Choomjinda U., Chen M.-M., Chen Y.-L., Wang H.-C. (2024). Mechanical Integrity of All-on-Four Dental Implant Systems: Finite Element Simulation of Material Properties of Zirconia, Titanium, and PEEK. Open Dent. J..

[37] Fiorillo L., Milone D., D’Andrea D., Santonocito D., Risitano G., Cervino G., Cicciù M. (2022). Finite Element Analysis of Zirconia Dental Implant. Prosthesis.

[38] Takahashi J.M.F.K., Dayrell A.C., Consani R.L.X., de Arruda Nóbilo M.A., Henriques G.E.P., Mesquita M.F. (2015). Stress Evaluation of Implant-Abutment Connections under Different Loading Conditions: A 3D Finite Element Study. J. Oral Implant..

[39] Reddy M.S., Sundram R., Eid Abdemagyd H.A. (2019). Application of Finite Element Model in Implant Dentistry: A Systematic Review. J. Pharm. Bioallied Sci..

[40] Geramizadeh M., Katoozian H., Amid R., Kadkhodazadeh M. (2017). Finite Element Analysis of Dental Implants with and without Microthreads under Static and Dynamic Loading. J. Long Term Eff. Med. Implant..

[41] (2016). Dentistry—Implants—Dynamic Loading Test for Endosseous Dental Implants. https://www.iso.org/standard/61997.html.

[42] TRI Dental Implants Int. AG Product Catalog 2025, Hünenberg, Switzerland. https://tri-implants.swiss/download/170926_en_product_catalogue_new_web.pdf.

[43] TRI Dental Implants Int. AG Matrix Line Product Catalog 2025, Hünenberg, Switzerland. https://tri-implants.swiss/download/2025-09-tri_-matrix-catalog-eu-01_web_1.pdf.

[44] (2022). Quantities and Units—Part 1: General. https://www.iso.org/standard/76921.html.

[45] Piotrowski B., Baptista A.A., Patoor E., Bravetti P., Eberhardt A., Laheurte P. (2014). Interaction of Bone-Dental Implant with New Ultra Low Modulus Alloy Using a Numerical Approach. Mater. Sci. Eng. C Mater. Biol. Appl..

[46] (2015). Implants for Surgery—Ceramic Materials Based on Yttria-Stabilized Tetragonal Zirconia (Y-TZP). https://www.iso.org/standard/62373.html.

[47] ASM Material Data Sheet. https://asm.matweb.com/search/specificmaterial.asp?bassnum=mtp641.

[48] Morgan Advanced Ceramics CIM Zirconia Technical Ceramic. https://www.matweb.com/search/datasheet.aspx?matguid=e90b81329aa547aa95433fc5dcb3940c&n=1&ckck=1.

[49] Chung S.M., Yap A.U.J., Koh W.K., Tsai K.T., Lim C.T. (2004). Measurement of Poisson’s Ratio of Dental Composite Restorative Materials. Biomaterials.

[50] Rho J.Y., Ashman R.B., Turner C.H. (1993). Young’s Modulus of Trabecular and Cortical Bone Material: Ultrasonic and Microtensile Measurements. J. Biomech..

[51] Helgason B., Perilli E., Schileo E., Taddei F., Brynjólfsson S., Viceconti M. (2008). Mathematical Relationships between Bone Density and Mechanical Properties: A Literature Review. Clin. Biomech..

[52] Wu D., Isaksson P., Ferguson S.J., Persson C. (2018). Young’s Modulus of Trabecular Bone at the Tissue Level: A Review. Acta Biomater..

[53] Prados-Privado M., Martínez-Martínez C., Gehrke S.A., Prados-Frutos J.C. (2020). Influence of Bone Definition and Finite Element Parameters in Bone and Dental Implants Stress: A Literature Review. Biology.

[54] Guan H., van Staden R., Loo Y.-C., Johnson N., Ivanovski S., Meredith N. (2009). Influence of Bone and Dental Implant Parameters on Stress Distribution in the Mandible: A Finite Element Study. Int. J. Oral Maxillofac. Implant..

[55] Frisardi G., Barone S., Razionale A.V., Paoli A., Frisardi F., Tullio A., Lumbau A., Chessa G. (2012). Biomechanics of the Press-Fit Phenomenon in Dental Implantology: An Image-Based Finite Element Analysis. Head Face Med..

[56] Shayanfard P., Tan X., Karl M., Wendler F. (2024). Finite Element Combined Design and Material Optimization Addressing the Wear in Removable Implant Prosthodontics. J. Funct. Biomater..

[57] Bozkaya D., Müftü S. (2004). Efficiency Considerations for the Purely Tapered Interference Fit (TIF) Abutments Used in Dental Implants. J. Biomech. Eng..

[58] Bulaqi H.A., Mousavi Mashhadi M., Safari H., Samandari M.M., Geramipanah F. (2015). Dynamic Nature of Abutment Screw Retightening: Finite Element Study of the Effect of Retightening on the Settling Effect. J. Prosthet. Dent..

[59] (2005). Fasteners—Torqueclamp Force Testing. https://www.studocu.com/row/document/sakarya-universitesi/mechatronic-engineering/iso-16047-2005-fasteners-torqueclamp-force-testing/6704899.

[60] Alemanno F., Peretti V., Tortora A., Spriano S. (2020). Tribological Behaviour of Ti or Ti Alloy vs. Zirconia in Presence of Artificial Saliva. Coatings.

[61] Torque Technical Data. https://www.tohnichi.com/torque-technical-data/.

[62] Kayumi S., Takayama Y., Yokoyama A., Ueda N. (2015). Effect of Bite Force in Occlusal Adjustment of Dental Implants on the Distribution of Occlusal Pressure: Comparison among Three Bite Forces in Occlusal Adjustment. Int. J. Implant Dent..

[63] Guven S., Atalay Y., Asutay F., Ucan M.C., Dundar S., Karaman T., Gunes N. (2015). Comparison of the Effects of Different Loading Locations on Stresses Transferred to Straight and Angled Implant-Supported Zirconia Frameworks: A Finite Element Method Study. Biotechnol. Biotechnol. Equip..

[64] Frost H.M. (2003). Bone’s Mechanostat: A 2003 Update. Anat. Rec. A Discov. Mol. Cell. Evol. Biol..

[65] Wang P.-S., Tsai M.-H., Wu Y.-L., Chen H.-S., Lei Y.-N., Wu A.Y.-J. (2023). Biomechanical Analysis of Titanium Dental Implants in the All-on-4 Treatment with Different Implant-Abutment Connections: A Three-Dimensional Finite Element Study. J. Funct. Biomater..

[66] Ravindran P.A., Raghavan R., Christopher K., Sramadathil S., George A., Sasi A.K. (2024). Stress Distribution by Parafunctional Loading on Tooth–Implant, Implant–Implant, and Tooth–Tooth-Supported Prosthesis: A Comparative Three-Dimensional Finite Element Analysis. J. Indian Prosthodont. Soc..

[67] Menacho-Mendoza E., Cedamanos-Cuenca R., Díaz-Suyo A. (2022). Stress Analysis and Factor of Safety in Three Dental Implant Systems by Finite Element Analysis. Saudi Dent. J..

[68] Sahoo N.R., Sahany S.K., Pandey V., Das A.C., Choudhury P., Panda S., Sahoo R. (2024). Finite Element Analysis of the Influence of Implant Tilting and the Direction of Loading on the Displacement and Micromotion of Immediately Loaded Implants. J. Pharm. Bioallied Sci..

[69] Quintas-Hijós J., Pérez-Pevida E. (2025). Influence of Intermediate Abutment Height and Timing of Placement on Marginal Bone Loss in Single Implant-Supported Crowns: A 12-Month Follow-up Randomized Clinical Trial. Clin. Oral Investig..

[70] Calatrava J., Sanz-Sánchez I., Molina A., Bollain J., Martín C., Sanz M. (2024). Effect of One-Time Placement of the Definitive Abutment versus Multiple Healing Abutment Disconnections and Reconnections during the Prosthetic Phase on Radiographic and Clinical Outcomes: A 12-Month Randomized Clinical Trial. Clin. Implant Dent. Relat. Res..

[71] D’Ercole S., Dotta T.C., Farani M.R., Etemadi N., Iezzi G., Comuzzi L., Piattelli A., Petrini M. (2022). Bacterial Microleakage at the Implant-Abutment Interface: An In Vitro Study. Bioengineering.

[72] Ruhstorfer M., Güth J.-F., Stimmelmayr M., Waltenberger L., Schubert O., Graf T. (2024). Systematic Review of Peri-Implant Conditions and Aesthetic Outcomes of Customized versus Conventional Healing Abutments. Int. J. Implant Dent..

[73] Galindo-Moreno P., León-Cano A., Monje A., Ortega-Oller I., O’Valle F., Catena A. (2016). Abutment Height Influences the Effect of Platform Switching on Peri-Implant Marginal Bone Loss. Clin. Oral Implant. Res..

[74] Vatėnas I., Linkevičius T. (2021). One Abutment One Time vs. Repeatable Abutment Disconnections in Implants, Restored with Cemented/Screw Retained Fixed Partial Dentures: Marginal Bone Level Changes. A Systematic Review and Meta-Analysis. Stomatologija.

[75] Sanz-Sánchez I., Molina A., Martin C., Bollain J., Calatrava J., Sanz M. (2024). The Effect of One-Time Abutment Placement on Clinical and Radiographic Outcomes: A 5-Year Randomized Clinical Trial. Clin. Oral Implant. Res..

[76] Montevecchi M., Valeriani L., Salvadori M.F., Stefanini M., Zucchelli G. (2025). Excess Cement and Peri-Implant Disease: A Cross-Sectional Clinical Endoscopic Study. J. Periodontol..

[77] Rasaie V., Abduo J., Falahchai M. (2022). Clinical and Laboratory Outcomes of Angled Screw Channel Implant Prostheses: A Systematic Review. Eur. J. Dent..

[78] Atieh M.A., Tawse-Smith A., Alsabeeha N.H.M., Ma S., Duncan W.J. (2017). The One Abutment-One Time Protocol: A Systematic Review and Meta-Analysis. J. Periodontol..

[79] Lv X.-L., Qian S.-J., Qiao S.-C., Gu Y.-X., Lai H.-C., Shi J.-Y. (2021). Clinical, Radiographic, and Immunological Evaluation of Angulated Screw-Retained and Cemented Single-Implant Crowns in the Esthetic Region: A 1-Year Randomized Controlled Clinical Trial. Clin. Implant Dent. Relat. Res..

[80] Warreth A., Doody K., Al-Mohsen M., Morcos O., Al-Mohsen M., Ibieyou N. (2015). Fundamentals of Occlusion and Restorative Dentistry. Part II: Occlusal Contacts, Interferences and Occlusal Considerations in Implant Patients. J. Ir. Dent. Assoc..

[81] (2024). Dentistry—Ceramic Materials. https://www.iso.org/standard/81718.html.

[82] Reis I.N.R.D., Reis I.N.R.D., Fukuoka G.L., Nagay B.E., Pannuti C.M., Spin-Neto R., da Silva E.V.F. (2025). Incidence of Peri-Implant Disease Associated with Cement- and Screw-Retained Implant-Supported Prostheses: A Systematic Review and Meta-Analysis. J. Prosthet. Dent..

[83] Barrett T. (1990). Fastener Design Manual.

[84] Kraus R.D., Espuelas C., Hämmerle C.H.F., Jung R.E., Sailer I., Thoma D.S. (2022). Five-year Randomized Controlled Clinical Study Comparing Cemented and Screw-retained Zirconia-based Implant-supported Single Crowns. Clin. Oral Implant. Res..

[85] Ionescu A., Marin M., Bayrich N., Fennema P., Nicolescu M.I., Jung R.E., Dodi A. (2025). Abutment-Free Tissue-Level Implants for Personalized Monolithic Zirconia Implant Crowns: A Retrospective Cohort Study. Int. J. Oral Maxillofac. Implant..

[86] Jung R.E., Sailer I., Hämmerle C.H.F., Attin T., Schmidlin P. (2007). In Vitro Color Changes of Soft Tissues Caused by Restorative Materials. Int. J. Periodontics Restor. Dent..

[87] Ferrari M., Carrabba M., Vichi A., Goracci C., Cagidiaco M.C. (2017). Influence of Abutment Color and Mucosal Thickness on Soft Tissue Color. Int. J. Oral Maxillofac. Implant..

[88] Totou D., Naka O., Mehta S.B., Banerji S. (2021). Esthetic, Mechanical, and Biological Outcomes of Various Implant Abutments for Single-Tooth Replacement in the Anterior Region: A Systematic Review of the Literature. Int. J. Implant Dent..

[89] Gehrke P., Zimmermann K.-P., Weinhold O., Dhom G., Fischer C., Sader R. (2021). Optical Efficacy of Titanium Nitride-Coated Abutment Material on Soft Tissue Discoloration: A Spectrophotometric In Vitro Analysis. Int. J. Oral Maxillofac. Implant..

